# ZSTK3744, a Novel Aryl Hydrocarbon Receptor Agonist, Exhibits Efficacy against Chemotherapy-Resistant Triple-Negative Breast Cancer

**DOI:** 10.1158/2767-9764.CRC-25-0119

**Published:** 2026-02-27

**Authors:** Takahiro Ohashi, Tsubasa Nagasaki, Kayoko Kawai-Asami, Kotomi Akatsuka, Yuko Nagata, Sayuri Terada, Shinsuke Hiramoto, Eika Higashi, Ryuichiro Ohshita, Mizuki Kuramochi, Makoto Furuya, Mai Todoroki, Hisashi Yoshimi, Keiko Fukushima

**Affiliations:** 1 Biosciences, Department of Drug Discovery, R&D Center, Zenyaku Kogyo Co., Ltd., Tokyo, Japan.; 2 Organic Synthesis, Department of Drug Discovery, R&D Center, Zenyaku Kogyo Co., Ltd., Tokyo, Japan.; 3 Pharmacology, Department of Drug Discovery, R&D Center, Zenyaku Kogyo Co., Ltd., Tokyo, Japan.; 4 Safety Evaluation, Department of Drug Discovery, R&D Center, Zenyaku Kogyo Co., Ltd., Tokyo, Japan.

## Abstract

Triple-negative breast cancer (TNBC) is an aggressive subtype of breast cancer characterized by a poor prognosis owing to the lack of therapeutic targets, including hormone receptors and HER2. Systemic chemotherapy remains the primary treatment option; however, its efficacy is limited by chemotherapy resistance, a substantial challenge in clinical management. The present study aimed to develop a novel anticancer drug that overcomes chemotherapy resistance in TNBC. First, chemotherapy-resistant MDA-MB-468 cells derived from TNBC were developed via long-term exposure to adriamycin or paclitaxel. Compared with parental cells, these resistant cells exhibited reduced sensitivity to paclitaxel, adriamycin, and eribulin in the viability assays. Various compounds were screened for their cell growth–inhibitory activity against these resistant cells to identify novel therapeutic agents; ZSTK3744 was a promising candidate and showed significant antitumor activity *in vitro* and *in vivo*. ZSTK3744 upregulated *CYP1A1*, *CYP1B1*, and *TIPARP* expression in MDA-MB-468 cells but exhibited no cell growth–inhibitory activity in aryl hydrocarbon receptor (AhR)–knockout cells, indicating that ZSTK3744 acts as an AhR agonist. Notably, ZSTK3744 demonstrated superior tumor inhibition and lower pulmonary toxicity in *ex vivo* and *in vivo* models than other AhR agonists. These results suggest that ZSTK3744 combines robust cell growth–inhibitory activity with a favorable safety profile. In conclusion, ZSTK3744 is a promising candidate for overcoming chemotherapy resistance in TNBC, addressing the urgent need for more effective treatment options for this aggressive cancer subtype.

**Significance:**

ZSTK3744 was identified through screening for its cell growth–inhibitory activity on chemotherapy-resistant TNBC cells. ZSTK3744 exerts its activity through AhR. ZSTK3744 exhibits superior cell growth–inhibitory activity compared with other AhR agonists and demonstrates a favorable safety profile. Therefore, ZSTK3744 is a promising therapeutic agent for overcoming chemotherapy resistance in TNBC.

## Introduction

Breast cancer is the most commonly diagnosed cancer in women and is characterized by substantial heterogeneity in its clinical and molecular features (1–3). Breast cancer is classified into subtypes based on the expression levels of the estrogen receptor, progesterone receptor, and HER2. Triple-negative breast cancer (TNBC), which lacks estrogen receptor, progesterone receptor, and HER2 expression, accounts for 15% to 20% of all breast cancer cases (3, 4). Owing to its aggressive nature and limited treatment options, TNBC is associated with a poorer prognosis than other breast cancer subtypes (3, 5).

Without targeted therapies, systemic chemotherapy remains the primary treatment for TNBC, particularly in neoadjuvant settings. Chemotherapeutic agents such as adriamycin, paclitaxel, and fluorouracil achieve a pathologic complete response (pCR) in only 10% to 30% of cases, and patients who achieve pCR have a favorable prognosis (6, 7). However, many patients who fail to achieve pCR often experience early recurrence and distant metastasis. Chemotherapeutic agents like eribulin and capecitabine are also used for relapse and refractory breast cancer cases; however, the objective response rate of these therapies remains limited (8). Furthermore, although innovative treatments such as immunotherapy, PARP inhibitors, and therapeutic antibodies have been approved for specific patient populations, their applicability is restricted by stringent eligibility criteria, leaving many patients without viable treatment options (9–15). For instance, the PARP inhibitor olaparib has been approved by the FDA for patients with HER2-negative breast cancer with germline *BRCA1* or *BRCA2* mutations. Olaparib has shown a significant improvement in overall survival (OS) and progression-free survival (PFS); however, the prevalence of *BRCA1* and *BRCA2* mutations in TNBC is only 11.1% to 12.4% and 1.9% to 4.3%, respectively (12, 13). Similarly, trastuzumab–deruxtecan (T-Dxd), a topoisomerase inhibitor–conjugated anti-HER2 antibody, has shown efficacy in HER2-low breast cancer. Approximately 36% of patients with TNBC are classified as HER2 low, suggesting that T-Dxd could improve OS and PFS in these patients (14, 15). However, most patients with TNBC are HER2 negative, limiting the therapeutic benefits of T-Dxd for the broader patient population. These challenges highlight the urgent and unmet clinical need for novel therapeutic approaches in TNBC.

Chemotherapy resistance, which is driven by mechanisms such as ATP-binding cassette (ABC) transporter upregulation, cancer stem cells, and evasion of apoptosis, remains a major cause of treatment failure in TNBC (16, 17). Specifically, the overexpression of P-glycoprotein, also known as ABC transporter subfamily B1 (ABCB1), contributes to chemotherapy resistance in various cancer types, including TNBC (18–20). ABCB1 mediates paclitaxel, docetaxel, adriamycin, eribulin, and olaparib resistance by effluxing these drugs out of ovarian and breast cancer cells (21–23). In breast cancer, increased ABCB1 expression following neoadjuvant therapy is associated with poorer prognoses (24). In addition to ABCB1, other subfamilies of ABC transporters, such as ABC sub-family C member 1, ABC sub-family C member 11 (ABCC11), and ABC sub-family G member 2, also contribute to chemotherapy resistance in breast cancer (25). Targeting drugs that are not recognized by ABC transporters represents a promising strategy to overcome chemotherapy resistance.

The aryl hydrocarbon receptor (AhR) is a transcription factor that regulates gene expression by binding to various ligands, including xenobiotics such as dioxins and benzo[a]pyrene, as well as endogenous molecules like kynurenine (26, 27). Upon ligand binding, AhR forms a heterodimer with the AhR nuclear translocator (ARNT), enabling its translocation to the nucleus. Within the nucleus, this complex binds to xenobiotic response element sequences, initiating the transcription of metabolism-related enzymes such as cytochrome P450 (CYP) 1A1 and CYP1A2, which facilitate xenobiotic degradation. Finally, AhR controls detoxification, immune responses, and carcinogenicity, highlighting its diverse and complex biological functions (26, 28, 29).

Recently, AhR has attracted attention as a therapeutic target for various diseases, including cancer, atopic dermatitis, and immunosuppressive diseases (30–33). Several clinically used agents and natural products, including omeprazole, flutamide, and indole-3-carbinol, have been identified as AhR modulators, underscoring the translational potential of AhR-targeted drug discovery (34). Particularly, AhR overexpression in various tumor types has been recognized as a promising tumor-specific therapeutic target (35, 36). In recent years, several novel anticancer agents targeting the AhR, including both agonists and antagonists, have been developed for various cancers; for example, BAY2416964, AFP464 (a prodrug of aminoflavone), and Phortress (a prodrug of 5F-203) have demonstrated marked antitumor activity in multiple cancer models (37–41). Notably, several of these compounds have shown promising efficacy in TNBC (42–45). However, development has been limited by pulmonary toxicity observed in early-phase trials of AFP464 and Phortress, both of which are AhR agonists (46, 47). Additionally, pulmonary toxicity of 5F-203 has also been demonstrated in human *ex vivo* lung tissue models (48). These findings indicate that AhR agonists need to retain their therapeutic potential while minimizing off-target effects.

To address chemotherapy resistance in TNBC, paclitaxel- and adriamycin-resistant TNBC cell lines were developed and screened to identify novel therapeutic agents. This study aimed to investigate the therapeutic potential of ZSTK3744 as a treatment for chemotherapy-resistant TNBC, focusing on its antitumor mechanisms and safety profile.

## Materials and Methods

### Chemical compounds

ZSTK3341 and ZSTK3744, which were synthesized by Zenyaku Kogyo Co., Ltd., were screened from our compound library. The methods for synthesizing ZSTK3341 and ZSTK3744 and their quality control details are described in Supplementary Fig. S1. Paclitaxel (T7402-25MG, Merck), adriamycin (HY-15142, Nacalai Tesque), and eribulin (Eisai) were used as standard chemotherapy agents for both *in vitro* and *in vivo* studies. Aminoflavone (NSUN-120919-4, BioDuro-Sundia), 5F-203 (SML0983-5MG, Merck), and its prodrug Phortress (15342, Cayman Chemical) were used as comparative AhR agonists. AFP464, an aminoflavone prodrug, was synthesized at Zenyaku Kogyo Co., Ltd. according to patent WO02084162. PI3K activity was measured using the PI3K HTRF Assay Kit (33-016, Merck) according to the manufacturer’s instructions. Recombinant human PI3K isoforms α (14-602, Merck), β (14-603, Merck), γ (14-558, Merck), and δ (14-604, Merck) were used as enzyme sources. Homogeneous time-resolved fluorescence (HTRF) was measured at 665 and 620 nm using a Wallac EnVision 2102 Multilabel Reader (Revvity). PI3K activity was expressed as the HTRF ratio (10,000 × 665 nm/620 nm). Enzyme activity was reported as a percentage of the untreated control.

### Cell lines

The breast cancer cell lines MDA-MB-468 (MM468 and HTB-132, RRID: CVCL_0419), DU4475 (HTB-123, RRID: CVCL_1183), T47D (HTB-133, RRID: CVCL_0553), and Hs578T (HTB-126, RRID: CVCL_0332) were obtained from the ATCC. MDA-MB-453 (MM453 and RCB1192, RRID: CVCL_0418) was obtained from the Riken BioResource Research Center. MM468, MM453, DU4475, and Hs578T were derived from patients with TNBC, whereas T47D was derived from a patient with luminal A subtype breast cancer. These cells were authenticated using short tandem repeat DNA profiling and tested for *Mycoplasma* using PCR by the vendors, and the cells were used at passages 5 to 20. MM468 and Hs578T cells were cultured in DMEM (11965-092, Thermo Fisher Scientific) supplemented with 10% FBS (10437028, Thermo Fisher Scientific) and 100 μg/mL kanamycin sulfate (15160054, Thermo Fisher Scientific) in an incubator at 37°C and 5% CO_2_. MM453 cells were cultured in Leibovitz’s L-15 medium (11415-064, Thermo Fisher Scientific) supplemented with 10% FBS and 100 μg/mL kanamycin sulfate in an incubator at 37°C and 0% CO_2_. DU4475 and T47D cells were cultured in RPMI-1640 (11875-093, Thermo Fisher Scientific) supplemented with 10% FBS and 100 μg/mL kanamycin sulfate in an incubator at 37°C and 5% CO_2_. MM468 cells were exposed to adriamycin (HY-15142, Nacalai Tesque) or paclitaxel (T7402-25MG, Merck) at concentrations of 10 to 140 nmol/L and 2 to 320 nmol/L, respectively, to establish drug-resistant cell lines. Surviving cells were designated as adriamycin-resistant (AR) and paclitaxel-resistant (PR) MM468 cells.

### Establishment of gene-modified breast cancer cells

T47D cells were simultaneously transfected with a commercially available vector encoding guide RNA and Cas9 for AhR (sc-400297, Santa Cruz Biotechnology), along with a vector encoding a homology-directed repair template and a puromycin resistance gene for AhR (sc-400297-HDR, Santa Cruz Biotechnology). Transfections were conducted via electroporation using the MaxCyte system (MaxCyte-STX, MaxCyte). After transfection, to select cells that had undergone homologous recombination with the template harboring a puromycin resistance sequence, the transfected cells were treated with puromycin (ant-pr-1, InvivoGen) at concentrations of 1 to 5 μg/mL for 5 days. Surviving cells were then single-cloned by limiting dilution and screened for the desired sequence. The presence of the AhR-knockout (KO) sequence was confirmed using the BigDye Terminator v3.1 Cycle Sequencing Kit (4337455, Thermo Fisher Scientific) and analyzed with a SeqStudio Genetic Analyzer (A35644, Thermo Fisher Scientific). Cells in which the homology-directed repair (HDR) template sequence was homozygously inserted into the AhR locus were designated as AhR-KO cells. Finally, the loss of AhR was confirmed by evaluating AhR protein expression through Western blot analysis. To create CYP1A1-KO cells, T47D cells were simultaneously transfected with a commercially available vector encoding guide RNA and Cas9 for *CYP1A1* (sc-400511, Santa Cruz Biotechnology), along with a vector encoding a homology-directed repair template and a puromycin resistance gene for *CYP1A1* (sc-400511-HDR-2, Santa Cruz Biotechnology). The sequence of CYP1A1-KO cells was verified using the same method employed for AhR. CYP1A1/1B1 activity was assessed using the P450-Glo CYP1A1 Assay System (V8751, Promega). CYP1A1/CYP1B1 activity was assessed based on chemiluminescence intensity, determined using a SpectraMax iD3 (Molecular Devices).

The *ABCB1* sequence was amplified from the cDNA of MM468 cells using PrimeSTAR MAX DNA Polymerase (R045A, TaKaRa) and cloned into the pcDNA 3.1 (+) vector (V79020, Thermo Fisher Scientific). MM468 cells were transfected with the *ABCB1*-cloned pcDNA3.1 vector by electroporation and exposed to geneticin (4727878001, Roche Diagnostics) at concentrations from 200 to 500 μg/mL. Surviving cells were single-cloned by limiting dilution, and ABCB1-high clones (#10 and #12) were identified using a flow cytometer.

### Cell growth assay

Cancer cells were incubated with the indicated drugs for 72 hours at 37°C. Cell viability was assessed using the Cell Counting Kit-8 (343-07623, Dojindo Laboratories), and absorbance was measured at 450 nm using a microplate reader (MTP-320, Corona Electric). Viability was calculated using the formula described below. CH223191 (C8124-5MG, Merck) and Z-VAD-FMK (FMK001, R&D Systems) were used as an AhR antagonist and a pan-caspase inhibitor, respectively.

$$\text{Cell}\;\text{viability}\;\left(\%\right)\;=\;\left({\text{OD}}_{450}\;\text{of}\;\text{tested}\;\text{sample}\;\text{after}\;\text{treatment}\;-\;{\text{OD}}_{450}\;\text{of}\;\text{medium}\right)/\left({\text{OD}}_{450}\;\text{of}\;\text{untreated}\;\text{sample}\;-\;{\text{OD}}_{450}\;\text{of}\;\text{medium}\right)\;\times \;100$$

### Western blotting

Cancer cells were harvested and homogenized in RIPA buffer containing 25 mmol/L Tris-HCl (207-06275, FUJIFILM Wako Pure Chemical Corp.), 150 mmol/L sodium chloride, 1% Nonidet P40 substitute (492016, Merck), 0.25% sodium deoxycholate (044-18812, FUJIFILM Wako Pure Chemical Corp.), 0.1% SDS, and 1% protease inhibitor (539134, Merck). Protein concentrations in the cell lysates were determined using the Pierce Bicinchoninic Acid Protein Assay Kit (23227, Pierce). SDS-PAGE sample buffer was added to 5 μg of protein from each lysate, and the samples were incubated at 95°C for 5 minutes. The prepared samples were subjected to SDS-PAGE using 4% to 20% gradient polyacrylamide gels and transferred to a nitrocellulose membrane (170-4158, Bio-Rad Laboratories). The membranes were blocked with 5% skim milk (198-10605, FUJIFILM Wako Pure Chemical Corp.) in TBS containing 0.05% Tween 20 (10 mmol/L Tris-HCl, 150 mmol/L sodium chloride, and 0.05% Tween 20; P7949-100ML, Merck). The membranes were incubated with an anti-AhR antibody (sc-133088, Santa Cruz Biotechnology, RRID: AB_2273721) or an anti-GAPDH antibody (5174, Cell Signaling Technology, RRID: AB_10622025) at the appropriate dilution, followed by incubation with a horseradish peroxidase–conjugated anti–rabbit IgG secondary antibody (#7074, Cell Signaling Technology, RRID: AB_2099233). Protein bands were visualized using an enhanced chemiluminescence reagent (RPN2232, Cytiva) and imaged with the LAS 4000 system (Cytiva).

### Flow cytometry

Cells were incubated with PE-conjugated anti-ABCB1 antibody (919405, BioLegend, RRID: AB_2629542) for 1 hour on ice. Fluorescence intensity was analyzed using a flow cytometer (CytoFLEX, Beckman Coulter, RRID: SCR_019627). For apoptotic cell analysis, cells were stained with annexin V and propidium iodide (PI; 15342-54, Nacalai Tesque) according to the manufacturer’s instructions. The sum of annexin V–positive cells (early apoptotic cells) and annexin V– and PI-positive cells (late apoptotic cells) was identified as apoptotic cells. Fluorescence signals were detected and analyzed using flow cytometry.

### Gene expression analysis

Total RNA was extracted from the breast cancer cells using the RNeasy Mini Kit (74104, Qiagen) according to the manufacturer’s instructions. RNA sequencing (RNA-seq) analysis of MM468/AR and MM468/PR cells was performed by Macrogen Japan Corp. DNA microarray of MM468 cells treated with ZSTK3744 was conducted by Filgen, Inc. The arrays used were Clariom S (Thermo Fisher Scientific). RNA quality was assessed using the Bioanalyzer 2100 (Agilent Technologies), confirming RNA quality. Data normalization was conducted using the robust multi-array average method. Data and statistical analyses were performed with the Microarray Data Analysis Tool (Filgen). The RNA-seq and microarray datasets have been deposited in the Gene Expression Omnibus under the following accession numbers: RNA-seq (GSE305008) and microarray (GSE305009). For reverse transcription, 1 μg of total RNA was converted into cDNA using the Transcriptor First Strand cDNA Synthesis Kit (04897030001, Roche Diagnostics) according to the manufacturer’s protocol. The cDNA was used as a template for real-time PCR analysis. The TaqMan probe and primer sets for *CYP1A1* (Hs01054797_g1), *CYP1B1* (Hs00164383_m1), *TIPARP* (Hs00296054_m1), *ABCB1* (Hs00184500_m1), and *18S rRNA* (Hs03003631_g1) were obtained from Thermo Fisher Scientific. The PCR reactions were performed using the LightCycler TaqMan Master Mix (04735536001, Roche Diagnostics) and the LightCycler 96 system (05815916001, Roche Diagnostics) according to the manufacturer’s instructions; 18S rRNA was used as the internal control for normalization.

### Surface plasmon resonance assay

The binding affinity of ZSTK3744 to AhR was assessed using surface plasmon resonance (SPR) technology on a Biacore T100 (Cytiva, RRID: SCR_019679). PBS (#9808, Cell Signaling Technology) containing 1% DMSO (031-24051, FUJIFILM Wako Pure Chemical Corp.) was used as the running buffer. The AhR peptide (RWVQSNARLIYRNGRPDYIIATQRPLTDEEK) was obtained from Thermo Fisher Scientific. The AhR peptide was immobilized as a ligand on the flow cells of a CM5 sensor chip (#29104988, Cytiva), followed by the injection of ZSTK3744 (0.61, 2.44, 9.77, 39.1, 156.3, and 625 nmol/L) as the analyte.

### Mouse xenograft model

NOD.CB17-*Prkdc*^*scid*^/J (NOD/SCID, RRID; IMSR_JAX:001303) mice (female, 6 weeks old) were obtained from The Jackson Laboratory Japan Inc. The animals were housed in animal facilities at the R&D center of Zenyaku Kogyo Co., Ltd., under specific pathogen-free (SPF) conditions. Tumor cells (5 × 10^5^) were suspended in Matrigel Basement Membrane Matrix (354234, Corning) and injected subcutaneously into the mice. For the MM468 models, tumor slices were transplanted from mouse to mouse. Each mouse was subcutaneously inoculated with a tumor fragment measuring 3 × 3 × 3 mm. Drug administration was initiated when tumor volumes reached 100 to 300 mm^3^. In the MM468/AR xenograft model, eribulin (Eisai) at 1.5 mg/kg and ZSTK3744 at 2.5 or 10 mg/kg were administered intravenously on days 0, 7, and 14. Tumor volume and body weight were measured from day 0 to 32 (mean ± SD, *n* = 5). In the parental MM468 xenograft model, ZSTK3744 was administered intravenously at 1, 2.5, or 5 mg/kg on days 0, 4, and 8 (mean ± SD, *n* = 5). In the MM453 xenograft model, ZSTK3744 was administered intravenously at 2.5, 5, or 10 mg/kg on the same schedule (mean ± SD, *n* = 5). Tumor volumes were measured in both models from day 0 to day 32. ZSTK3744 was dissolved in a 5% glucose solution. The vehicle control group received injections of the 5% glucose solution. The length and width of the subcutaneous tumor mass were measured with calipers in live mice, and the tumor volume was calculated using the following formula:

$$\text{Tumor}\;\text{volume}\;\left({\text{mm}}^{3}\right)\;=\;\left(\text{length}\;\times \;{\text{width}}^{2}\right)/2$$

### Toxicologic studies

The study of precision-cut lung slices (PCLS) was conducted at the Institute for *In Vitro* Sciences, Inc. (IIVS). In the proliferation assay, PCLS was continuously treated with ZSTK3744, AFP464, and Phortress. The culture medium and drugs were replaced on days 3 and 6. PCLS viability was assessed using the Cell Counting Kit-8 on day 7 (mean ± SD, *n* = 4). Frozen blocks of PCLS were shipped from IIVS to our laboratory for further analysis. Total RNA was extracted from PCLS using the MagMAX mirVana Total RNA Isolation Kit (A27828, Thermo Fisher Scientific) according to the manufacturer’s instructions. Analysis of *CYP1A1* mRNA levels was performed using the LightCycler system.

Female 6-week-old Crl:CD rats (RRID: RGD_734476) were purchased from The Jackson Laboratory Japan Inc. The animals were housed in the animal facilities at the R&D center of Zenyaku Kogyo Co., Ltd. under SPF conditions. After 1 week of acclimatization, the rats were intravenously administered ZSTK3744 at doses of 25 and 50 mg/kg. The administrations were repeated once daily for four consecutive days. The rats in the vehicle control group received 5% glucose solution injections. Tissues were fixed in a Tissue-Tek Ufix (5985, Sakura Finetek) solution, dehydrated, and embedded in paraffin. Deparaffinized sections, cut to a thickness of 3 μm, were stained with hematoxylin and eosin for histopathologic examination.

Toxicologic studies on dogs were conducted at the BoZo Research Center, Inc. A female and a male beagle, aged 25 to 34 months, were used in a dose-escalation study of ZSTK3744. Doses of 0.38, 0.96, 2.4, 6, and 15 mg/kg (7.68, 19.2, 48, 120, and 300 mg/m^2^) of ZSTK3744 were administered intravenously on days 0, 7, 14, 21, and 28, respectively. Blood was collected before the third administration (2.4 mg/kg) and at 1, 2, and 4 hours after administration. After inducing hemolysis by adding water, the leukocyte-enriched fraction was obtained, the samples were washed twice with PBS, and total RNA was extracted from the cells. Seven days after the final administration, the lungs and liver of the dogs were histopathologically examined. A dose of 11.4 mg/kg (228 mg/m^2^) of AFP464 was administered once a week to one dog; respiratory abnormalities were observed the day after the second dose, prompting a necropsy. Deparaffinized sections, cut to a thickness of 3 μm, were stained with hematoxylin and eosin for histopathologic examination.

### Statistical analysis

Data are presented as the mean ± SD. The Mann–Whitney U test was used to compare two groups. Multiple comparisons were conducted using one-way ANOVA with a Dunnett multiple comparison test or the Kruskal–Wallis test with the Steel–Dwass method. Statistical significance was determined using Excel Statistics ver.7 (ESUMI). Differences were considered significant when the *P* value was < 0.05.

### Ethics statement

All animal experiments were performed following protocols approved by the Animal Experimental Investigations Committee at Zenyaku Kogyo Co., Ltd. (approval numbers: 19-45A, 21-23, and 21-27). This facility is certified by the Japan Pharmaceutical Information Center. *In vivo* experiments were performed in accordance with the ARRIVE guidelines.

## Results

### Establishment of chemotherapy-resistant cells

**Figure 1.**
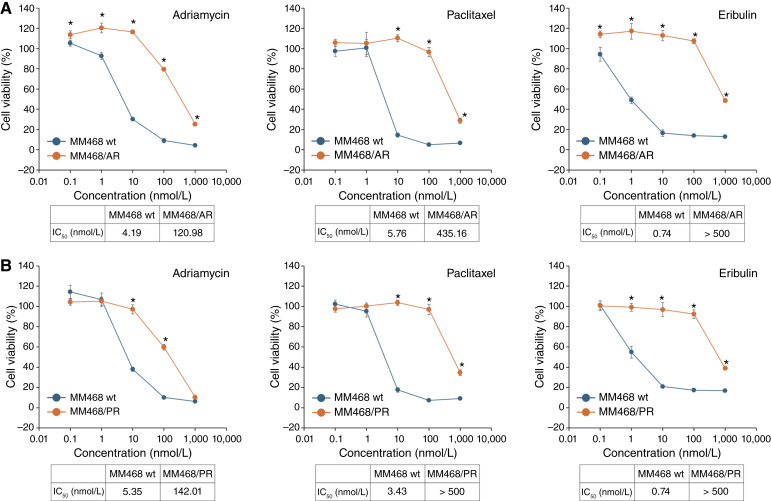
Establishment of chemotherapy-resistant TNBC cells. MM468/AR and MM468/PR cell lines were established. Parental MM468 cells, along with the MM468/AR (**A**) or MM468/PR (**B**) cells, were treated with adriamycin, paclitaxel, or eribulin at the indicated concentrations for 72 hours. Cell viability was assessed using the Cell Counting Kit-8 assay, and the results are presented as mean ± SD of quadruplicate experiments. Statistical analysis was performed using the Mann–Whitney U test (*, *P* < 0.05 vs. parental MM468). Parental MM468 cells are presented in blue, whereas chemotherapy-resistant MM468 cells (MM468/AR and MM468/PR) are presented in orange. wt, wild type.

Paclitaxel- and adriamycin-resistant MM468 cells were established to develop antitumor agents for chemotherapy-resistant TNBC. The IC_50_ for adriamycin-induced cell growth inhibition in MM468/AR was 120.98 nmol/L, which was notably higher than the 4.19 nmol/L observed in the parental cells (Fig. 1A), indicating resistance in MM468/AR cells. Furthermore, MM468/AR cells exhibited markedly decreased sensitivity to paclitaxel and eribulin, suggesting multidrug resistance. Similarly, the IC_50_ of paclitaxel in MM468/PR cells exceeded 500 nmol/L compared with 3.43 nmol/L in parental cells (Fig. 1B). MM468/PR cells were resistant to paclitaxel, adriamycin, and eribulin.

RNA-seq analysis in MM468, MM468/AR, and MM468/PR cells was performed to elucidate the mechanisms underlying chemotherapy resistance. A total of 436 and 932 genes were significantly upregulated, whereas 540 and 815 genes were downregulated in MM468/AR and MM468/PR cells, respectively, compared with parental MM468 cells. Several genes related to chemoresistance were identified among these differentially expressed genes, including ABC transporters, nicotinamide N-methyltransferase, and tumor protein 63 (Supplementary Tables S1 and S2; refs. 49, 50). Given the multidrug resistance observed in these cells, ABC transporters were focused upon, which mediate drug resistance in cancer (18–25). ABCC11 expression was elevated 5.78-fold in MM468/PR cells; however, it was not included in the top 30 upregulated genes listed in Supplementary Table S1. Notably, ABCB1 upregulation was observed in both MM468/AR and MM468/PR cells, with a 572.5- and 534.5-fold change, respectively (Supplementary Tables S1 and S2). These findings strongly suggest that ABCB1 upregulation contributes to the chemotherapy resistance observed in these cell lines. This result was validated by analyzing *ABCB1* mRNA using real-time PCR and assessing ABCB1 protein expression on the cell surface via flow cytometry (Supplementary Fig. S2A and S2B). Therefore, ABCB1-overexpressing cells were generated to investigate whether ABCB1 contributes to resistance to chemotherapeutic agents. ABCB1 expression levels were upregulated in clones #10 and #12 compared with parental MM468 cells (Supplementary Fig. S2C). The cell growth–inhibitory activity of paclitaxel, adriamycin, and eribulin was significantly reduced in both clone #10 and #12 compared with parental MM468 cells (Supplementary Fig. S2D–S2F). Similarly, docetaxel, vinorelbine, and epirubicin activity was diminished in clone #10 (Supplementary Fig. S3). These results suggest that ABCB1 plays a major role in chemotherapeutic resistance in MM468/AR and MM468/PR cells.

### Identification of ZSTK3744 as an antitumor agent for chemotherapy-resistant TNBC

**Figure 2.**
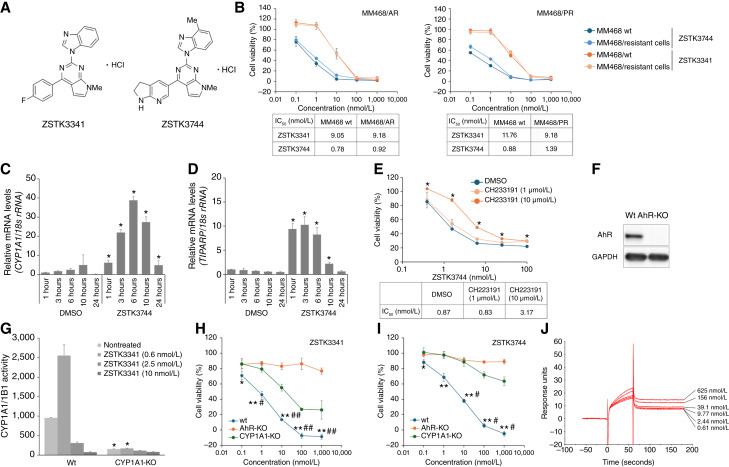
Screening of antitumor agents for chemotherapy-resistant TNBC cells. **A,** The chemical structures of ZSTK3744 and ZSTK3341, which were screened from our compound library for their cell growth–inhibitory activity against chemotherapy-resistant cells. **B,** Parental MM468, MM468/AR, and MM468/PR cells were treated with ZSTK3744, ZSTK3341, adriamycin, paclitaxel, or eribulin at the indicated concentrations for 72 hours. Cell viability was assessed using the Cell Counting Kit-8 assay (mean ± SD of quadruplicate experiments). The mRNA levels of *CYP1A1* (**C**) and *TIPARP* (**D**) were measured by qRT-PCR using cDNA synthesized from total RNA extracted from MM468 cells treated with DMSO or ZSTK3744. Data are normalized to control genes 18S rRNA and presented as mean ± SD from quadruplicate experiments. Statistical analysis was performed using the Mann–Whitney U test (*, *P* < 0.05 vs. DMSO for all time points). **E,** MM468 cells were treated with ZSTK3744 or in combination with the AhR antagonist CH223191 (1 and 10 μmol/L) for 72 hours, and cell viability was assessed using a Cell Counting Kit-8 assay (mean ± SD of quadruplicate experiments). Statistical analysis was performed using one-way ANOVA, followed by a Dunnett test (*, *P* < 0.05 vs. DMSO for all concentrations). **F,** AhR and GAPDH protein expression levels were analyzed using Western blotting in parental T47D (wild type) and AhR-KO T47D cells. GAPDH was used as a loading control. **G,** CYP1A1 and CYP1B1 activity induced by ZSTK3341 at the indicated dose in CYP1A1-KO T47D and parental cells. Enzyme activity was assessed using the P450-Glo CYP1A1 Assay System. CYP1A1/CYP1B1 activity was measured based on chemiluminescence intensity, which was determined using SpectraMax iD3. The results are presented as mean ± SD of quadruplicate experiments. Statistical analysis was performed using the Mann–Whitney U test (*, *P* < 0.05 vs. parental T47D cells). **H** and **I,** T47D wild type (blue), CYP1A1-KO T47D (green), and AhR-KO T47D (orange) cells were treated with ZSTK3341 (**H**) and ZSTK3744 (**I**) at the indicated concentrations for 72 hours, and cell viability was assessed using a Cell Counting Kit-8 assay (mean ± SD of quadruplicate experiments). Statistical analysis was performed using the Mann–Whitney U test (*, *P* < 0.05; **, *P* < 0.01 vs. respective CYP1A1-KO T47D cells at each concentration; ^#^*P* < 0.05, ^##^*P* < 0.01 vs. respective AhR-KO T47D cells at each concentration). **J,** AhR peptide was captured on the sensor chip, followed by ZSTK3744 injection at the indicated concentration. wt, wild type.

To identify effective antitumor agents with potent activity against chemotherapy-resistant cells, we screened our compound library using cell viability assays. The PI3K inhibitor ZSTK474 was previously identified in our laboratory, which led to the establishment of a structurally diverse library of aromatic heterocyclic compounds (51–53). In the present study, we screened a library of approximately 1,500 aromatic heterocyclic compounds, focusing on IC_50_ values for tumor growth–inhibitory activity as the primary selection criterion. Initially, parental MM468 cells were treated with the compounds, and cell viability was measured after 72 hours. Compounds with IC_50_ values lower than 10 nmol/L were selected. In the secondary screening, we assessed the growth-inhibitory activity against chemotherapy-resistant cells, selecting compounds that exhibited comparable efficacy with the parental cells. Ultimately, ZSTK3341 (compound **1**, IC_50_ = 2.89 nmol/L) emerged as the lead candidate because of its potent effect on parental MM468 cells and comparable IC_50_ values in chemotherapy-resistant cells (Fig. 2A and B; Table 1). Subsequently, the structure of ZSTK3341 was optimized to improve its cell growth–inhibitory activity. Additionally, ZSTK3341 was deemed unsuitable as a drug candidate because of its poor aqueous solubility and limited solubility in most organic solvents, which complicated its synthetic handling. To address these limitations, we implemented a solubility screening strategy alongside the screening based on cell growth–inhibitory activity. Initial modifications focused on the pyrrole condensed with pyrimidine. Substituting pyrrole with furan or pyridine resulted in reduced cell growth–inhibitory activity, yielding compound **2** (IC_50_ = 14.82 nmol/L) and compound **3** (IC_50_ = 14.02 nmol/L), respectively, with no improvement in solubility (Table 1). Furthermore, substituting the nitrogen of pyrrole with an ethyl group decreased cell growth–inhibitory activity (compound **4,** IC_50_ = 11.09 nmol/L). The 4-fluorophenyl moiety was then targeted for structural optimization. Replacing it with various five- and six-membered heteroaromatic rings generally preserved cell growth–inhibitory activity. Notably, pyridine derivatives exhibited potent activity and improved solubility (compounds **5**–**8**). These compounds possess highly planar structures, leading us to expect improved solubility with the introduction of sp^3^ carbon. Upon examining substituents containing sp^3^ carbon, we found that derivatives with saturated rings, in particular, demonstrated superior activity and solubility (compounds **9** and **10**). To further enhance activity, we optimized the benzimidazole moiety. Modifying this moiety showed that methyl substitution enhanced activity; compound **11** (IC_50_ = 3.27 nmol/L) displayed substantially greater cell growth–inhibitory activity than compound **9** (IC_50_ = 5.59 nmol/L). The application of this activity-enhancing modification to compound **10** led to the successful development of ZSTK3744 (compound **12**, IC_50_ = 0.87 nmol/L), which shows both potent cell growth–inhibitory activity and favorable physicochemical properties, achieving approximately a 3.3-fold improvement in cell growth–inhibitory activity compared with the lead compound ZSTK3341. Notably, ZSTK3744 demonstrated comparable inhibitory effects across MM468/PR, MM468/AR, and parental MM468 cells (Fig. 2B). Importantly, ZSTK3341 and ZSTK3744 do not exhibit PI3K-inhibitory activity, unlike ZSTK474 (Supplementary Fig. S4).

**Table 1.** Structure optimization of ZSTK3341.

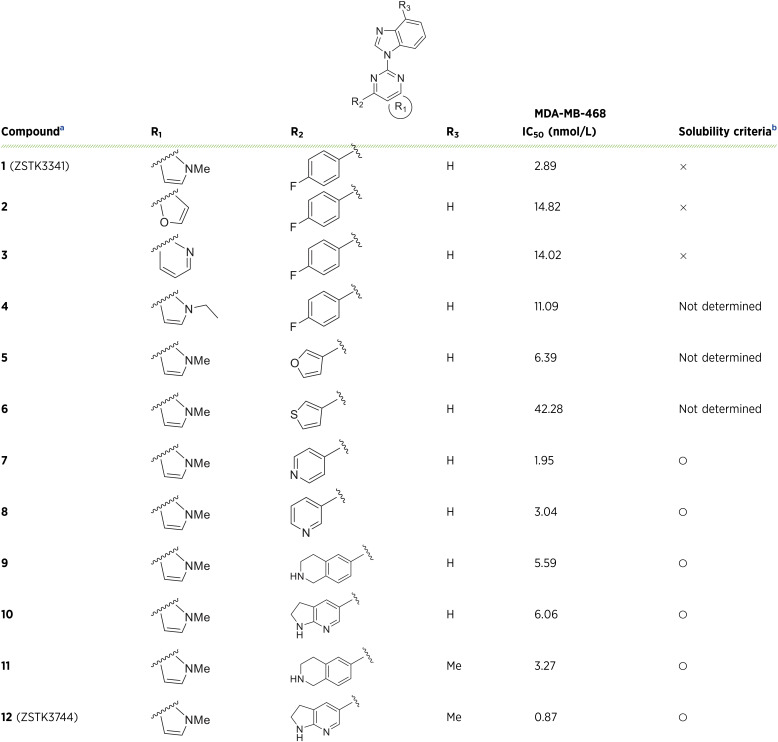

IC_50_ values for cell growth–inhibitory activity were evaluated using the Cell Counting Kit-8 assay.

a Except for compound **2**, all compounds were synthesized as hydrochloride salts and used in both cell growth–inhibitory and solubility assays.

b Solubility was visually assessed in 5% glucose solution: ○ indicates dissolution at 0.5 mg/mL, whereas × indicates suspension at 0.5 mg/mL.

DNA microarray was performed on MM468 cells following a 6-hour treatment with ZSTK3744 to identify the target molecules of ZSTK3744. Genes with altered expression are listed in Table 2. Pathway enrichment analysis using the Kyoto Encyclopedia of Genes and Genomes database revealed significant enrichment of the chemical carcinogenesis pathway after ZSTK3744 treatment. Among the upregulated genes identified in the transcriptome analysis, *CYP1A1* (Fig. 2C) and *TIPARP* (Fig. 2D) showed increased mRNA expression levels, which were further validated using real-time PCR. These findings suggest that ZSTK3744 enhances the AhR pathway. To determine whether AhR is involved in the cell growth–inhibitory activity of ZSTK3744, MM468 cells were treated with ZSTK3744 in the presence of the AhR antagonist CH223191. Co-treatment with CH223191 reduced ZSTK3744 activity in a dose-dependent manner (Fig. 2E). Additionally, AhR-KO and CYP1A1-KO T47D cells were generated using the CRISPR/Cas9 system. AhR deficiency in these cells was confirmed via Western blotting (Fig. 2F). Immunoblotting could not be performed as CYP1A1 was not observed in T47D cells under unstimulated conditions. Therefore, enzyme activity following AhR stimulation was used as a marker for CYP1A1 KO (Fig. 2G). The activity may still be detected because this assay cross-reacts with CYP1B1. However, CYP1A1 activity was reduced, suggesting that CYP1A1 is functionally deficient. AhR-KO cells exhibited resistance to the cell growth–inhibitory activity of ZSTK3341 and ZSTK3744 (Fig. 2H and I), and the activity of these compounds was reduced in CYP1A1-KO cells. These results indicate that AhR and CYP1A1 are essential for the cell growth–inhibitory activity of ZSTK3744. The direct binding ability of ZSTK3744 with AhR peptide was confirmed using SPR (Fig. 2J). Response levels increased in a dose-dependent manner with ZSTK3744 (0.61, 2.44, 9.77, 39.1, 156, and 625 nmol/L). These results show that ZSTK3744 binds directly to AhR, indicating that ZSTK3744 is an AhR agonist.

**Table 2.** Top 20 significantly upregulated and downregulated differentially expressed genes after treatment with ZSTK3744 compared with DMSO-treated cells.

Upregulation			Downregulation		
Gene symbol	Gene accession	Log_2_ ratio	Gene symbol	Gene accession	Log_2_ ratio
*CYP1A1*	NM_000499	7.80	*HIST1H1D*	NM_005320	−4.10
*ATF3*	NM_001030287	4.25	*HIST1H2BM*	NM_003521	−4.03
*HBEGF*	NM_001945	4.21	*STARD13*	NM_001243466	−3.52
*CYP1B1*	NM_000104	3.93	*HIST3H2BB*	NM_175055	−3.33
*STC2*	NM_003714	3.70	*IKZF2*	NM_001079526	−3.17
*DLX1*	NM_001038493	3.65	*TNS3*	NM_022748	−3.12
*TIPARP*	NM_001184717	3.38	*PSRC1*	NM_001005290	−2.97
*ID2*	NM_002166	3.34	*ROR1*	NM_001083592	−2.83
*SNAI1*	NM_005985	3.24	*MAML2*	NM_032427	−2.80
*PMAIP1*	NM_021127	3.13	*BMP2*	NM_001200	−2.55
*RND1*	NM_014470	3.10	*HIST1H2AB*	NM_003513	−2.49
*SHISA2*	NM_001007538	3.08	*GPR39*	NM_001508	−2.46
*CHAC1*	NM_001142776	2.92	*TGFBR3*	NM_001195683	−2.38
*RHOB*	NM_004040	2.87	*TOX*	NM_014729	−2.36
*MAFF*	NM_001161572	2.85	*HIST1H3G*	NM_003534	−2.36
*SGK1*	NM_001143676	2.84	*RUNX2*	NM_001015051	−2.35
*DDIT3*	NM_001195053	2.78	*FIGN*	NM_018086	−2.29
*PRDM1*	NM_001198	2.74	*RPTOR*	NM_001163034	−2.27
*FOSL1*	NM_001300844	2.67	*KIF18A*	NM_031217	−2.23
*FAM46C*	NM_017709	2.65	*HIST1H2BG*	NM_003518	−2.22

Total RNA was extracted from MM468 cells treated with DMSO or ZSTK3744 (1 μmol/L) to evaluate the effect of ZSTK3744 treatment on gene expression. Comprehensive mRNA expression changes were assessed using DNA microarray analysis. Filgen, Inc. conducted the analysis.

AhR agonists, such as aminoflavone and 5F-203, induce cell growth inhibition in breast cancer cells through apoptosis (40, 41, 45, 54, 55). Therefore, the involvement of apoptosis in the cell growth inhibition mechanism of ZSTK3744 was investigated. The percentage of apoptotic cells was assessed after a 24-hour ZSTK3744 treatment with annexin V and PI staining. Treatment with paclitaxel as a positive control and ZSTK3744 significantly increased the percentage of apoptotic cells compared with DMSO-treated controls (Supplementary Fig. S5A and S5B). Pretreatment with pan-caspase inhibitor, Z-VAD-FMK, attenuated ZSTK3744-induced apoptosis (Supplementary Fig. S5C and S5D). In addition, Z-VAD-FMK attenuated the cell growth–inhibitory activity of ZSTK3744 in a dose-dependent manner (Supplementary Fig. S5E). These findings demonstrate that ZSTK3744, like other AhR agonists, exerts its cell growth–inhibitory activity through an apoptosis-mediated mechanism.

### Comparison of the cell growth–inhibitory activity of ZSTK3744 and chemotherapeutic agents in chemotherapy-resistant cells

**Figure 3.**
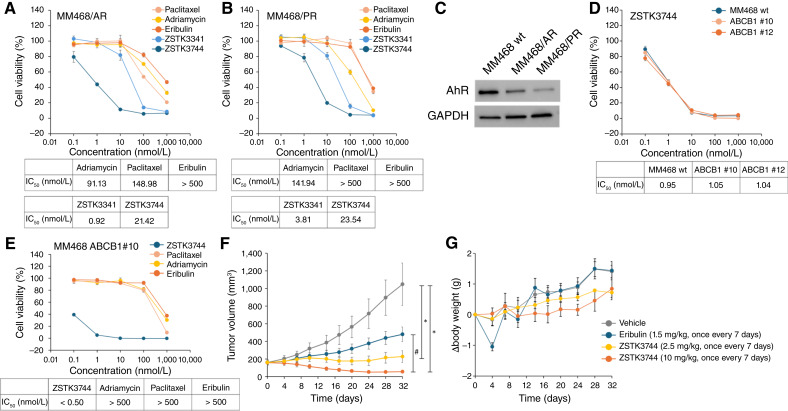
Evaluation of the cell growth–inhibitory activity of ZSTK3744 on chemotherapy-resistant cells. Parental MM468, MM468/AR (**A**), and MM468/PR cells (**B**) were treated with ZSTK3744, ZSTK3341, adriamycin, paclitaxel, or eribulin at the indicated concentrations for 72 hours. Cell viability was assessed using the Cell Counting Kit-8 assay (mean ± SD of quadruplicate experiments). **C,** AhR and GAPDH protein expression levels were analyzed using Western blotting in parental MM468, MM468/AR, and MM468/PR cells. GAPDH was used as a loading control. **D,** Parental and ABCB1-overexpressed MM468 cell clone #10 and #12 were treated with ZSTK3744 for 72 hours. Cell viability was assessed using the Cell Counting Kit-8 assay (mean ± SD of quadruplicate experiments). **F,** ABCB1-overexpressed MM468 cell clone #10 were treated with ZSTK3744, adriamycin, paclitaxel, and eribulin for 72 hours. Cell viability was assessed using the Cell Counting Kit-8 assay (mean ± SD of quadruplicate experiments). **E,** Evaluation of the antitumor activity of ZSTK3744 and eribulin in a mouse xenograft model. NOD/SCID mice were subcutaneously injected with MM468/AR cells. Administration of these drugs was initiated when the tumor volume reached 100–300 mm^3^. ZSTK3744 was administered intravenously at doses of 2.5 or 10 mg/kg, whereas eribulin was administered intravenously at a dosage of 1.5 mg/kg on days 0, 7, and 14. **G,** Tumor volumes and body weight were measured on days 0, 4, 7, 10, 14, 17, 20, 24, 28, and 32 (mean ± SD, *n* = 5). Body weight data are presented as the change from baseline (day 0). Statistical analysis was performed using the Kruskal–Wallis test, followed by the Steel–Dwass test (*, *P* < 0.05 vs. vehicle-treated group; ^#^*P* < 0.05 vs. eribulin-treated group on day 32 of administration). wt, wild type.

The growth-inhibitory activity of ZSTK3341, ZSTK3744, and chemotherapeutic agents used for TNBC treatment was evaluated in MM468/AR and MM468/PR cells. ZSTK3744 and ZSTK3341 demonstrated more potent efficacy than adriamycin, paclitaxel, and eribulin (Fig. 3A and B). Furthermore, the cell growth–inhibitory activity of ZSTK3744 was more potent than that of gemcitabine, carboplatin, capecitabine, and docetaxel in MM468/AR cells (Supplementary Fig. S3). AhR expression levels were downregulated in chemotherapy-resistant cells (Fig. 3C).

Owing to the contribution of high ABCB1 expression to chemotherapeutic resistance, the effect of ABCB1 overexpression on ZSTK3744 efficacy was evaluated. ZSTK3744 exhibited comparable cell growth–inhibitory activity in parental MM468 and MM468/ABCB1 clone #10 and #12 cells (Fig. 3D). In addition, ZSTK3744 activity was more potent than that of adriamycin, paclitaxel, eribulin, gemcitabine, carboplatin, capecitabine, and docetaxel in MM468/ABCB1 #10 cells (Fig. 3E; Supplementary Fig. S3). These results indicate that although ABCB1 overexpression induces resistance to chemotherapeutic agents, ZSTK3744 retains its efficacy regardless of ABCB1 overexpression.

A mouse xenograft model was used to further evaluate the efficacy of ZSTK3744. The antitumor effects were assessed based on tumor volume measurements. ZSTK3744 inhibited tumor growth in a dose-dependent manner, with this effect persisting until day 32 (Fig. 3F). In contrast, eribulin demonstrated limited efficacy (Fig. 3F). Notably, neither treatment significantly affected the body weight of the mice, indicating minimal systemic toxicity (Fig. 3G). These results suggest that ZSTK3744 is a potent antitumor agent against chemotherapy-resistant tumors.

### Evaluation of the antitumor activity of ZSTK3744 on TNBC cells *in vitro* and *in vivo*

**Figure 4.**
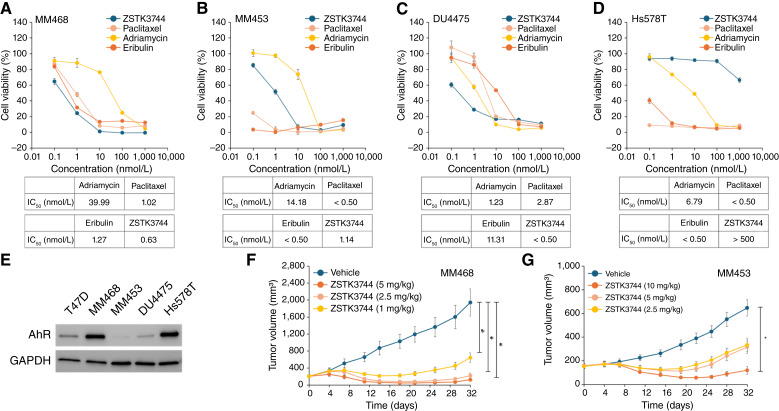
Evaluation of the antitumor activity of ZSTK3744 on various TNBC cell lines. MM468 (**A**), MM453 (**B**), DU4475 (**C**), and Hs578T (**D**) cells were treated with ZSTK3744, paclitaxel, adriamycin, or eribulin at the indicated concentrations for 72 hours. Cell viability was assessed using the Cell Counting Kit-8 assay (mean ± SD of quadruplicate experiments). **E,** AhR and GAPDH protein expression levels were analyzed using Western blotting in wild-type T47D, MM468, MM453, DU4475, and Hs578T cells. GAPDH was used as a loading control. **F** and **G,** Evaluation of the antitumor activity of ZSTK3744 was conducted in mouse xenograft models. NOD/SCID mice were subcutaneously implanted with slices of tumors from MM468 (**F**) and injected with MM453 cells mixed with Matrigel (**G**). Drug administration was initiated when the tumor volume reached 100–300 mm^3^. ZSTK3744 was administered intravenously at doses of 1, 2.5, and 5 mg/kg for MM468 and 2.5, 5, and 10 mg/kg for MM453 on days 0, 4, and 8. Tumor volumes for MM468 and MM453 were measured from day 0 to day 32 (mean ± SD, *n* = 5). Statistical analysis was performed using the Kruskal–Wallis test, followed by the Steel–Dwass test (*, *P* < 0.05 vs. vehicle-treated group on day 32 of administration).

TNBC cell lines, including MDA-MB-453 (MM453), DU4475, and Hs578T, and MM468 were treated with ZSTK3744 and standard chemotherapy agents for 72 hours to evaluate the cell growth–inhibitory activity of ZSTK3744 on TNBC. ZSTK3744 significantly reduced the viability of MM468 (Fig. 4A), MM453 (Fig. 4B), and DU4475 cells (Fig. 4C). However, it did not significantly affect the viability of Hs578T cells (Fig. 4D). The potency of cell growth–inhibitory activity induced by ZSTK3744 and chemotherapy agents varied among TNBC cell lines. The AhR expression levels were evaluated in these cell lines (Fig. 4E). No clear correlation was observed between AhR expression levels and the cell growth–inhibitory activity of ZSTK3744. For example, although Hs578T cells exhibited high AhR expression levels, they showed limited sensitivity to ZSTK3744.

Mouse xenograft models of parental MM468 and MM453 cells were established to further assess the antitumor activity of ZSTK3744 *in vivo*. Mice were administered ZSTK3744 at the indicated doses on days 0, 4, and 8, and tumor volumes were measured from day 0 to day 32 (Fig. 4F and G). In MM468-transplanted mice, ZSTK3744 administration at 2.5 and 5 mg/kg significantly reduced the tumor volume compared with pretreatment values on day 18 (*P* < 0.05 vs. day 0). Similarly, in MM453-transplanted mice, ZSTK3744 administration at 10 mg/kg reduced the tumor volume to below the pretreatment levels on day 19 (*P* < 0.05 vs. day 0). These results demonstrate the robust antitumor activity of ZSTK3744 *in vitro* and *in vivo*, highlighting its potential as a therapeutic agent for TNBC.

### Comparison of the efficacy and toxicity of ZSTK3744 and other AhR agonists *in vitro*

**Figure 5.**
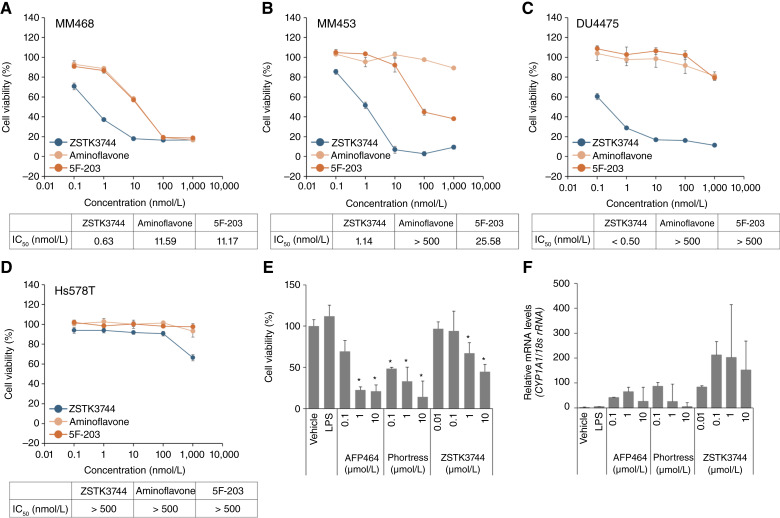
Comparison of cell growth–inhibitory activity and toxicity of ZSTK3744 and other AhR agonists. MM468 (**A**), MM453 (**B**), DU4475 (**C**), and Hs578T cells (**D**) were treated with ZSTK3744, aminoflavone, or 2-(4-amino-3-methylphenyl)-5-fluorobenzothiazole (5F-203) at the indicated concentrations for 72 hours, and cell viability was assessed using the Cell Counting Kit-8 assay (mean ± SD of quadruplicate experiments). **E,** PCLS were continuously treated with ZSTK3744 (0.01, 0.1, 1, and 10 μmol/L), AFP464 (0.1, 1, and 10 μmol/L), or Phortress (0.1, 1, and 10 μmol/L) for 7 days. Cell viability was measured using the Cell Counting Kit-8 assay (mean ± SD of triplicate experiments). Statistical analysis was performed using one-way ANOVA, followed by a Dunnett test (*, *P* < 0.01). **F,***CYP1A1* mRNA levels after treatment with ZSTK3744, AFP464, or Phortress were quantified using qRT-PCR. Data from PCLS experiments were generated at the IIVS. LPS, lipopolysaccharide.

The cell growth–inhibitory activity of ZSTK3744 was compared with that of other AhR agonists in TNBC cell lines, including parental MM468, MM453, DU4475, and Hs578T (Fig. 5A–D). ZSTK3744 inhibited cell growth, and its effect was more potent than that of aminoflavone and 5F-203 in MM468, MM453, and DU4475 cells. Notably, aminoflavone exhibited no effects in DU4475 or Hs578T cells, in which ZSTK3744 was also inactive. These findings suggest that ZSTK3744 has broader and more potent cell growth–inhibitory activity than other AhR agonists.

Human PCLS was used to compare the potential effects of ZSTK3744 with other AhR agonists on lung tissue (48, 56). Although all compounds reduced the viability of PCLS, AFP464 and Phortress, the prodrugs of aminoflavone and 5F-203, respectively, exhibited greater toxicity at lower concentrations than ZSTK3744 (Fig. 5E). For instance, after treatment with 0.1 μmol/L of ZSTK3744, AFP464, and Phortress, the cell viability of PCLS was 94%, 69%, and 48%, respectively. Notably, ZSTK3744 had little effect on viability; however, it induced a stronger upregulation of *CYP1A1* mRNA than AFP464 and Phortress, suggesting a distinct mechanism of action (Fig. 5F). These results indicate that ZSTK3744 has low toxicity compared with other AhR agonists while maintaining superior cell growth–inhibitory activity.

To investigate the selectivity of ZSTK3744, we assessed the ability of ZSTK3744 to induce *CYP1A1* or *CYP1B1* mRNA expression. ZSTK3744 induced *CYP1A1* and *CYP1B1* mRNA expression in MM468, MM453, and DU4475 cells, in which it was active, whereas induction was minimal in Hs578T cells, in which the compound showed no activity (Supplementary Fig. S6A and S6B). Furthermore, ARNT isoform 3 may serve as an indicator of AhR agonist activity as the relative balance between ARNT isoform 1 and isoform 3 has been reported to regulate both the magnitude and qualitative direction of AhR-dependent transcriptional responses (57, 58). In addition, ARNT isoform 3 has been described as a biomarker of aminoflavone sensitivity in US20130177904A1. *ARNT* isoform 3 mRNA expression levels were higher in MM468, MM453, and DU4475 cells than in Hs578T cells (Supplementary Fig. S6C and S6D).

### Toxicologic studies in rats and dogs

**Figure 6.**
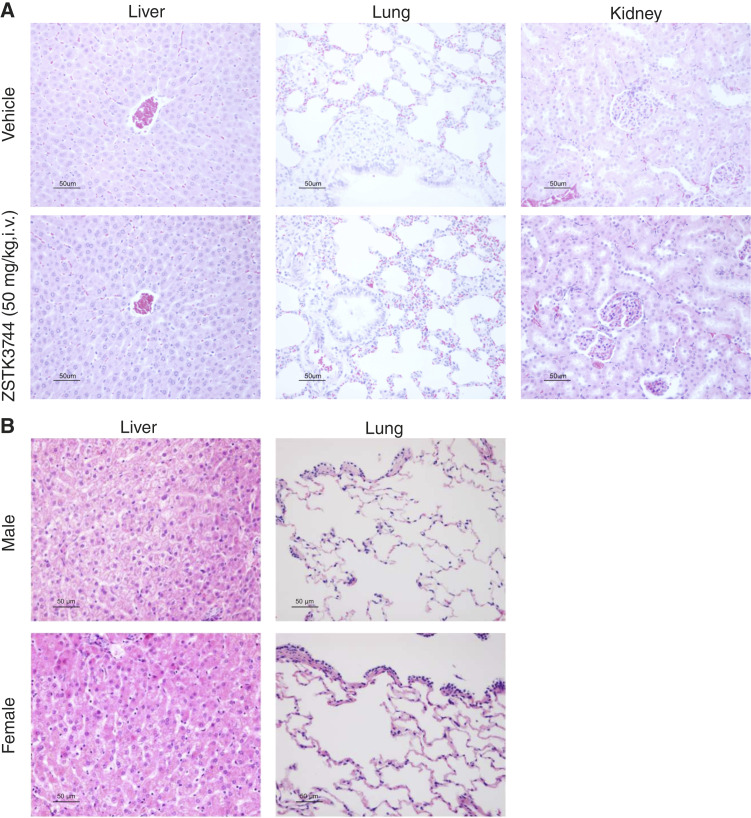
Evaluating the toxicity of ZSTK3744 *in vivo*. **A,** Representative hematoxylin and eosin staining images of rat lung, liver, and kidney following daily intravenous treatment with vehicle or ZSTK3744 (50 mg/kg) for four consecutive days. These images were analyzed to assess histologic changes associated with potential toxicity. **B,** Representative hematoxylin and eosin staining images of dog liver and lungs after intravenous administration of 0.38, 0.96, 2.4, 6, and 15 mg/kg (7.68, 19.2, 48, 120, and 300 mg/m^2^) of ZSTK3744 on days 0, 7, 14, 21, and 28, respectively. Seven days after the final administration, the lungs and liver of the dogs were histopathologically examined.

A toxicologic study in rats was performed to evaluate the systemic toxicity of ZSTK3744. Hypoactivity was observed after each administration but resolved within 30 minutes. The lungs, liver, and kidneys of the rats were histopathologically examined the day after the final administration; however, no histologic abnormalities were observed (Fig. 6A). These results indicate that ZSTK3744 is well tolerated and exhibits low toxicity compared with other AhR agonists while maintaining superior antitumor efficacy.

When AFP464 was administered to a dog at a dosage of 11.4 mg/kg (228 mg/m^2^) once a week, respiratory abnormalities occurred the day after the second dose, prompting a necropsy. Gross examination revealed dark reddish discoloration of the lungs. Furthermore, the histopathologic examination showed alveolar epithelial regeneration characterized by hypertrophy of type II alveolar epithelial cells, bronchial inflammation, and reactive bronchial epithelial hyperplasia (Supplementary Fig. S7A and S7B). These findings indicate that AFP464 induces pulmonary toxicity, highlighting dogs as a suitable animal model for assessing such toxic effects. Subsequently, a dose-escalation study in a male and a female dog was conducted to assess the pulmonary and hepatic toxicity of ZSTK3744 in detail. Doses of 0.38, 0.96, 2.4, 6, and 15 mg/kg of ZSTK3744 were administered intravenously on days 0, 7, 14, 21, and 28, respectively. Seven days after the final administration, the lungs and liver of the dogs were histopathologically examined. Mild Kupffer and immune cell infiltration was observed in the liver of the female dog; however, no histologic abnormalities were found in the lungs (Fig. 6B). No abnormalities were observed in the liver or lungs of the male dog. The induction of *CYP1A1* expression in the leukocyte-enriched fraction isolated by hypotonic red blood cell lysis after the third administration indicates that ZSTK3744 is pharmacologically active in dogs (Supplementary Fig. S7C). Although transient decreases in both white blood cell count and food intake were observed, these findings suggest that ZSTK3744 is well tolerated.

## Discussion

ZSTK3341 was identified as a compound with cell growth–inhibitory activity against cell lines established in our laboratory to develop therapeutic agents for patients with chemotherapy-resistant TNBC, for whom treatment options remain limited. These cell lines were resistant to adriamycin, paclitaxel, and eribulin, which are commonly used chemotherapeutic agents in the treatment of TNBC. Based on these findings, ZSTK3744 was subsequently developed to improve the growth-inhibitory activity and solubility of ZSTK3341. The present study evaluated the antitumor activity of ZSTK3744 against TNBC cells and assessed its toxicity profile.

Long-term exposure to adriamycin or paclitaxel resulted in the development of drug-resistant MM468 cell lines (MM468/AR and MM468/PR), which were resistant to adriamycin, paclitaxel, and eribulin. Transcriptome analysis revealed alterations in several genes associated with chemotherapy resistance, including members of the ABC transporter family. Notably, ABCB1 mRNA and protein expression levels were significantly upregulated in both cell lines. These findings suggest that ABCB1 plays a central role in the resistance mechanisms of MM468/AR and MM468/PR cells. ZSTK3341 and ZSTK3744 demonstrated potent cell growth–inhibitory activity against MM468/AR and MM468/PR cells, surpassing the efficacy of standard chemotherapeutic agents such as adriamycin, epirubicin, paclitaxel, docetaxel, eribulin, vinorelbine, and gemcitabine. ZSTK3744 activity was comparable between parental, resistant MM468, and ABCB1-overexpressing MM468 cells. Furthermore, ZSTK3744 inhibited tumor growth in a mouse xenograft model using chemotherapy-resistant cells, whereas eribulin demonstrated limited efficacy. These findings indicate that ZSTK3744 has potential as a therapeutic agent for chemotherapy-resistant TNBC.

DNA microarray analysis revealed that ZSTK3744 enriched the chemical carcinogenesis pathway, including xenobiotic-related mechanisms. Real-time PCR analysis confirmed that ZSTK3744 induced mRNA expression of *CYP1A1* and *TIPARP*. Additionally, the growth-inhibitory activity of ZSTK3744 was abolished in AhR-KO T47D cells, indicating that this activity is mediated through AhR. SPR assays further demonstrated that ZSTK3744 directly binds to AhR. These findings suggest that ZSTK3744 functions as an AhR agonist. However, as the SPR assay was performed using an AhR-derived peptide, the accuracy of detecting direct binding remains somewhat uncertain. Therefore, further *in vitro* and *in vivo* studies are required to confirm whether ZSTK3744 indeed acts as an AhR agonist.

AhR plays a pivotal role in detoxification and immune responses and is considered a therapeutic target for various diseases, including cancer, allergies, and autoimmune disorders (28–31). For instance, tapinarof, a topical AhR agonist, has been approved for the treatment of atopic dermatitis (33). In cancer, AhR agonists such as aminoflavone and 5F-203 have shown efficacy in preclinical studies (38–41). Notably, ZSTK3744 exhibited superior cell growth–inhibitory activity compared with aminoflavone and 5F-203 in MM468, MM453, and DU4475 cells. These findings suggest that ZSTK3744 has more potent antitumor efficacy than other AhR agonists. Similar to aminoflavone and 5F-203, ZSTK3744 induces cell growth inhibition through apoptosis (40, 41, 45, 54, 55), suggesting that its cytotoxic mechanism is shared with other AhR agonists. Although histologic and IHC data were not obtained in the present study, limiting the evidence provided in this regard, future studies involving histologic and IHC analyses of xenograft tumor sections, including assessments of AhR expression, apoptosis, and cellular proliferation, will be essential for further understanding the mechanisms responsible for the antitumor activity of ZSTK3744. Aminoflavones are metabolized by CYPs, for which expression is induced by AhR activation, into active forms that exert antitumor activity (40). Similarly, ZSTK3744 activity was reduced in CYP1A1-deficient cells, suggesting that ZSTK3744 may also require metabolic activation by CYP1A1. Although ZSTK3744 completely lost its activity in AhR-deficient cells, partial activity was still observed in CYP1A1-deficient cells. These differences suggest that, in addition to CYP1A1, other metabolic enzymes may contribute to ZSTK3744 activation.

Given that high AhR expression has been reported in various tumors, we examined the relationship between the cell growth–inhibitory activity of ZSTK3744 and AhR expression levels. Although MM468 cells exhibited higher AhR expression compared with MM453 and DU4475 cells, no remarkable differences were observed in their IC_50_ values. In addition, Hs578T cells, despite their high AhR expression, did not respond to ZSTK3744. Interestingly, AhR expression levels were downregulated in chemotherapy-resistant cells compared with parental MM468 cells; however, the cell growth–inhibitory activity of ZSTK3744 remained similar between the parental and chemotherapy-resistant MM468 cells. These findings indicate that AhR expression levels are not clearly correlated with the degree of ZSTK3744 activity. On the other hand, ZSTK3744 completely lost its activity in AhR-deficient cells, suggesting that the presence of AhR is essential for its activity. Taken together, these results suggest that although AhR is required for the activity of ZSTK3744, the degree of its activity is not solely determined by AhR expression levels and that other factors may be involved.

Clarifying the mechanism underlying the selectivity of ZSTK3744 is essential not only for understanding the mechanism of its activity but also for identifying patients who are most likely to benefit from this treatment. ZSTK3744 induces *CYP1A1* or *CYP1B1* mRNA expression in cells in which it exhibits cell growth–inhibitory activity, whereas induction of these CYPs is minimal in cells in which ZSTK3744 shows no activity. Induction of CYP expression may, therefore, serve as a biomarker of the pharmacologic activity of ZSTK3744. The expression of AhR target genes is regulated by ARNT, a dimerization partner of AhR. In particular, the ratio between ARNT isoforms 1 and 3 is critical for regulating gene expression (57, 58). One study has suggested that the expression of the isoform 3 variant of ARNT plays a crucial role in determining the sensitivity of aminoflavone; however, the precise mechanism remains to be elucidated as disclosed in US20130177904A1. In the present study, *ARNT* isoform 3 mRNA levels were higher in cells in which ZSTK3744 demonstrated activity than in cells in which it showed no activity. These results suggest that ARNT isoform 3 expression contributes to the selectivity of ZSTK3744, similar to aminoflavone. Aminoflavone is initially metabolized by CYPs and subsequently activated by sulfotransferases to generate its active metabolite (40). This finding raises the possibility that ZSTK3744 may also require a second metabolic step mediated by other enzymes and that the expression patterns of these enzymes could affect its tumor selectivity. Therefore, identifying predictive biomarkers for ZSTK3744 activity and toxicity and validating their reliability remain important challenges for future investigation.

AhR is highly expressed in the lungs (59, 60), raising concerns about potential pulmonary toxicity associated with AhR agonists. Several AhR agonists induce pulmonary toxicity in both *ex vivo* models and clinical trials (46–48). A cell viability assay using PCLS demonstrated that ZSTK3744, AFP464, and Phortress commonly induced cytotoxicity. However, ZSTK3744-induced cytotoxicity occurred at higher concentrations than the other AhR agonists. ZSTK3744 exhibited efficacy at approximately 1 nmol/L in TNBC cell lines. In comparison, cytotoxicity in PCLS was observed at approximately 1,000 nmol/L, indicating a 1,000-fold therapeutic margin, which is considered sufficient. Acute toxicity studies in rats revealed transient hypoactivity after ZSTK3744 administration, along with quick recovery and the absence of histopathologic abnormalities in the liver, lungs, or kidneys. These results indicate that ZSTK3744 is well tolerated. However, because no severe pulmonary toxicity has been observed with aminoflavone (61), it is possible that pulmonary toxicity cannot be accurately assessed in rodents. Because reports on aminoflavone have shown that dogs are highly sensitive to pulmonary toxicity (54), and the present study showed that AFP464 induced pulmonary toxicity, a preliminary toxicity study of ZSTK3744 was also conducted in dogs. To investigate whether ZSTK3744 induces AhR activation, we examined the expression of AhR downstream targets in leukocytes from dogs used in the toxicity studies and observed increased *CYP1A1* mRNA levels. Our findings suggest that ZSTK3744 activates AhR signaling *in vivo*, even in nontumor tissues, thereby functioning as a systemic inducer of AhR activation. However, no abnormalities were observed following the dose escalation of ZSTK3744 from 0.38 to 15 mg/kg, except for mild immune cell infiltration in the liver and transient decreases in white blood cell count and food intake. The relationship between immune cell infiltration and drug administration remains unclear. A large-scale toxicity study in dogs is required to more precisely assess the pulmonary and hepatic toxicity of ZSTK3744. Finally, careful monitoring will be essential during clinical trials. Although AhR activation has been implicated in both tumor-promoting and tumor-suppressive processes, selective AhR modulators (SAhRM) can induce context-dependent transcriptional responses without exhibiting carcinogenicity (42, 62–64). ZSTK3744 displays several features consistent with SAhRMs, including the selective induction of the AhR target genes *CYP1A1* and *CYP1B1* in responsive cells, metabolic activation that may lead to active metabolites, and a wide therapeutic margin between effective and toxic concentrations. The biological outcomes of AhR activation depend on multiple factors, such as ligand structure, receptor affinity, co-regulator expression, metabolic activation, and the extent of receptor activation. Therefore, elucidating whether ZSTK3744 functions as an SAhRM that preferentially activates tumor-suppressive pathways while minimizing toxic or tumor-promoting responses will be crucial for its clinical development.

In conclusion, ZSTK3744 demonstrates potential as a therapeutic agent for chemotherapy-resistant TNBC. It has robust antitumor activity in both chemotherapy-resistant and -sensitive TNBC cell lines, *in vitro* and *in vivo*. Notably, its efficacy is not compromised by common chemotherapy resistance mechanisms, such as ABC transporter overexpression. Furthermore, ZSTK3744 shows superior and broader antitumor activity than aminoflavone and 5F-203. Toxicity studies revealed that ZSTK3744 is well tolerated, with favorable therapeutic margins. These results position ZSTK3744 as a promising candidate for patients with limited treatment options, including those who have relapsed after standard chemotherapy. However, to fully address these unmet needs, further preclinical and clinical investigations are warranted to comprehensively assess its safety and therapeutic potential.

## Supplementary Material

Supplementary Figure S1 Synthesis of compounds 1–12

Supplementary Figure S2 Investigation of the effects of ABCB1 on chemotherapy resistance

Supplementary Figure S3 Evaluation of cell growth-inhibitory effects of standard chemotherapy agents on resistant and ABCB1-overexpressing cells

Supplementary Figure S4 Evaluation of PI3K enzyme activity

Supplementary Figure S5 Analysis of apoptotic cells

Supplementary Figure S6 Evaluation of biomarkers for ZSTK3744

Supplementary Figure S7 Evaluating the toxicity of AhR agonists in vivo

Supplementary Table S1 Top 30 significantly upregulated and downregulated differentially expressed genes in MM468/AR cells compared to parental cells

Supplementary Table S2 Top 30 significantly upregulated and downregulated differentially expressed genes in MM468/PR cells compared to parental cells

## Data Availability

The data presented in the current study were generated at Zenyaku Kogyo Co., Ltd. The data are available from the corresponding author on reasonable request. The RNA-seq and microarray datasets generated and analyzed during this study have been deposited in the Gene Expression Omnibus under the accession numbers GSE305008 for RNA-seq and GSE305009 for microarray.
