# Supplementary material for: ZSTK3744, a Novel Aryl Hydrocarbon Receptor Agonist, Exhibits Efficacy against Chemotherapy-Resistant Triple-Negative Breast Cancer
Source: Cancer Res Commun. 2026 Feb 27;6(2):421–36. doi: 10.1158/2767-9764.CRC-25-0119 (PMC13148475; doi:10.1158/2767-9764.CRC-25-0119)
Supplement: Supplementary Figure S1 — Synthesis of compounds 1–12 [file crc-25-0119_supplementary_figure_s1_suppsf1.docx]

**Supplementary Fig. S1. Synthesis of compounds 1–12**

**General**

The reactions were performed under an argon atmosphere, and reaction temperatures were measured externally. Their actions were monitored using thin-layer chromatography on precoated silica gel (60F_254_) glass plates (0.25 mm) from Merck (Rahway, NJ, USA) and visualized using UV light (254 nm). ^1^H and ^13^C NMR spectra were measured using a JNM-ECS400 or JNM-ECZ400S spectrometer (JEOL Ltd., Tokyo, Japan), and the analysis was performed using Delta software 6.0.0 (JEOL Ltd.). Chemical shifts are in parts per million. For ^1^H NMR, tetramethylsilane (TMS) served as the internal standard (δ). The spectral data are reported as follows: chemical shift, integration, multiplicity (s = singlet, d = doublet, t = triplet), and coupling constant in Hertz. For ^13^C NMR, TMS was used as an internal standard. Mass spectrometry detection was APCI.

***General procedure for Suzuki–Miyaura cross-coupling reaction (GP1)***

Dichloride (1 eq.) and boronic acid or boronic acid ester (1 eq. – 1.5 eq.) were mixed.

Case A: The reactants were suspended in dioxane (0.25 M relative to the dichloride), and 1 N Na₂CO₃ aq. (0.8 eq. – 2.0 eq.) and PdCl₂(PPh₃)₂ (0.07 eq. – 0.1 eq.) were added. The mixture was stirred under reflux for 1 h. After cooling, the mixture was extracted with dichloromethane and purified using silica gel column chromatography.

Case B: The reactants were suspended in dimethyl sulfoxide (0.25 M relative to the dichloride), and K₂CO₃ (1.3 eq.), H₂O (0.5 M with respect to the dichloride), and Pd(PPh₃)₄ (0.014 eq.) were added, and the mixture was stirred at 70 °C for 1 h. After cooling, the mixture was extracted with dichloromethane and purified using silica gel column chromatography.

Case C: Reactants were suspended in dioxane (0.25 M relative to the dichloride), and K₂CO₃ (1.3 eq.–1.5 eq.), H₂O (0.5 M relative to the dichloride), and PdCl₂(PPh₃)₂ (0.02 eq.-0.1 eq.) or Pd(PPh_3_)_4_ (0.02 eq.-0.1 eq.), and heated at 160 °C for 5 min using a microwave synthesis apparatus. After cooling, the mixture was extracted with dichloromethane and purified using silica gel column chromatography.

***General procedure for Buchwald–Hartwig cross-coupling reaction (GP2)***

Case A: Chloride (1 eq.) and benzimidazole or benzimidazole derivative (1.0 eq.-1.3 eq.) were suspended in dimethylformamide (DMF) (0.2 M relative to the chloride), followed by the addition of Cs₂CO₃ (1.3 eq - 1.5 eq.), XPhos (0.1 eq.), Pd₂(dba)₃ (0.02 eq - 0.05 eq.), and the mixture was stirred at 130 °C for 1 h. After cooling, the mixture was extracted with dichloromethane and purified using silica gel column chromatography.

Case B: Chloride (1 eq.) and benzimidazole (1.2 eq. – 1.5 eq.) were suspended in toluene (0.2 M relative to the chloride), followed by the addition of K_3_PO_4_ (1.5 eq. – 2.0 eq.), 2-(Di-tert-butylphosphino)-3,4,5,6-tetramethyl-2′,4′,6'-triisopropyl-1,1′-biphenyl (0.01 eq.), and Pd₂(dba)₃ (0.02 eq - 0.1 eq.), and the mixture was stirred at 120 °C for 3 h. After cooling, the mixture was extracted with dichloromethane and purified using silica gel column chromatography.

Case C: Chloride (1 eq.) and benzimidazole (1.1 eq.) were suspended in DMF (0.3 M relative to the chloride), followed by the addition of Cs₂CO₃ (1.3 eq.), XPhos (0.1 eq.), and Pd₂(dba)₃ (0.05 eq.), and the mixture was stirred at 160 °C for 10 min using a microwave synthesis apparatus. After cooling, the mixture was extracted with dichloromethane and purified using silica gel column chromatography.

***General procedure for hydrochloride salt of compounds (GP3)***

HCl/dioxane solution (4 N; 0.2 M – 0.9 M relative to the chloride) was added to respective compounds (1 eq.) in MeOH (0.05 M relative to the chloride) and stirred at room temperature for 1 h. After the solvent was removed under reduced pressure, ethyl acetate was added to the residue, and the resulting solid was filtered.

**Synthesis of ZSTK3341**

**2,4-Dichloro-7-methyl-7*H*-pyrrolo[2,3-*d*]pyrimidine (S1)**

A solution of 2,4-dichloro-7*H*-pyrrolo[2,3-*d*]pyrimidine (2.10 g, 11.1 mmol) in DMF (55 mL) was cooled to 0 ºC. K_2_CO_3_ (2.30 g, 16.7 mmol), and MeI (0.837 mL, 13.4 mmol) were added to this solution and stirred for 1 h. After adding H_2_O (200 mL) to the reaction solution, the precipitated solid was filtered and dried to produce **S1** as a white solid (2.11 g, 10.4 mmol, 94% yield).

^1^H-NMR (CDCl_3_) δ 3.85 (3H, s), 6.59 (1H, d, J = 3.7 Hz), 7.18 (1H, d, J = 3.7 Hz).

**2-Chloro-4-(4-fluorophenyl)-7-methyl-7*H*-pyrrolo[2,3-*d*]pyrimidine (S2)**

The preparation of **S2** was performed according to GP1A, using **S1** (800 mg, 3.96 mmol), 4-fluorophenylboronic acid (554 mg, 3.96 mmol), 1 N Na_2_CO_3_ aq. (3.20 mL, 3.20 mmol), and PdCl_2_(PPh_3_)_2_ (278 mg, 0.396 mmol). Further purification was performed using column chromatography (hexane:AcOEt = 1:2) to afford **S2** (900 mg, 3.44 mmol, 87% yield) as a white solid.

^1^H-NMR (CDCl_3_) δ 3.88 (3H, s), 6.77 (1H, d, J = 3.7 Hz), 7.20–7.25 (3H, m), 8.11–8.14 (2H, m).

**2-(1*H*-Benzo[*d*]imidazole-1-yl)-4-(4-fluorophenyl)-7-methyl-7*H*-pyrrolo[2,3-*d*]pyrimidine (S3)**

The preparation of **S3** was performed according to GP2A, using **S2** (67.4 mg, 0.257 mmol), benzimidazole (36.5 mg, 0.309 mmol), Cs_2_CO_3_ (126 mg, 0.385 mmol), XPhos (12.3 mg, 0.0257 mmol), and Pd_2_(dba)_3_ (4.70 mg, 0.00513 mmol). Further purification was performed using column chromatography (NH, hexane:AcOEt = 1:1) to afford **S3** (56.8 mg, 0.165 mmol, 64% yield) as a white solid.

^1^H-NMR (CDCl_3_) δ 4.00 (3H, s), 6.84 (1H, d, J = 3.7 Hz), 7.25–7.36 (3H, m), 7.37–7.50 (2H, m), 7.90 (1H, d, J = 7.8 Hz), 8.24–8.32 (2H, m), 8.81 (1H, d, J 0 = 8.2 Hz), 9.30 (1H, s).

**2-(1*H*-Benzo[*d*]imidazole-1-yl)-4-(4-fluorophenyl)-7-methyl-7*H*-pyrrolo[2,3-*d*]pyrimidine hydrochloride (ZSTK3341, Compound 1)**

The preparation of ZSTK3341 was performed according to GP3, using **S3** (56.0 mg, 0.163 mmol), 4 N HCl/dioxane solution (0.5 mL), and MeOH (3 mL). ZSTK3341 (45.4 mg) was obtained as a white solid.

^1^H-NMR (CD_3_OD) δ 4.05 (3H, s), 7.08 (1H, d, J = 3.7 Hz), 7.35-7.43 (2H, m), 7.69 (1H, d, J = 3.7 Hz), 7.71–7.85 (2H, m), 7.94 (1H, d, J = 8.2 Hz), 8.38–8.45 (2H, m), 9.11 (1H, d, J = 8.2 Hz), 10.46 (1H, s).

**Synthesis of Compound 2**

**2-Chloro-4-(4-fluorophenyl)flo[3,2-*d*]pyrimidine (S4)**

The preparation of **S4** was performed according to GP1C, using 2,4-dichlorofluoro[3,2-*d*]pyrimidine (82.4 mg, 0.436 mmol), 4-fluorophenylboronic acid (58.5 mg, 0.480 mmol), K_2_CO_3_ (90.3 mg, 0.654 mmol), and PdCl_2_(PPh_3_)_2_ (6.10 mg, 0.00874 mmol). Further purification was performed using column chromatography (hexane:AcOEt = 2:1) to afford **S4** (80.3 mg, 0.323 mmol, 74% yield) as a white solid.

^1^H-NMR (CDCl_3_) δ 7.01 (1H, d, J = 2.2 Hz), 7.24–7.30 (2H, m), 8.10 (1H, d, J = 2.2 Hz), 8.52–8.60 (2H, m).

**2-(1H-benzo[*d*]imidazol-1-yl)-4-(4-fluorophenyl)flo[3,2-*d*]pyrimidine (Compound 2)**

The preparation of Compound **2** was performed according to GP2A, using **S4** (78.7 mg, 0.293 mmol), benzimidazole (36.5 mg, 0.309 mmol), Cs_2_CO_3_ (126 mg, 0.385 mmol), XPhos (12.3 mg, 0.0258 mmol), and Pd_2_(dba)_3_ (4.70 mg, 0.00513 mmol). Further purification was performed using column chromatography (NH, hexane:AcOEt = 1:1) to afford Compound **2** (47.4 mg, 0.143 mmol, 49% yield) as a white solid.

^1^H-NMR (CDCl_3_) δ 7.16 (1H, d, J = 2.4 Hz), 7.33–7.42 (3H, m), 7.42–7.49 (1H, m), 7.74 (1H, d, J = 8.2 Hz), 8.41 (1H, d, J = 2.4 Hz), 8.60–8.66 (2H,m), 8.69 (1H, d, J = 8.2 Hz), 9.23 (1H,s).

**Synthesis of** **Compound 3**

**2-Chloro-4-(4-fluorophenyl)pyrido[2,3-*d*]pyrimidine (S5)**

The preparation of **S5** was performed according to GP1A, using 2,4-dichloropyridine[2,3-*d*]pyrimidine (250 mg, 1.25 mmol), 4-fluorophenylboronic acid (183 mg, 1.31 mmol), 1 N Na_2_CO_3_ aq. (2.20 mL, 2.20 mmol), and PdCl_2_(PPh_3_)_2_ (87.7 mg, 0.125 mmol). Further purification was performed using column chromatography (dichloromethane:MeOH = 12:1) to afford **S5** (301 mg, 1.16 mmol, 93% yield) as a white solid.

^1^H-NMR (CDCl_3_) δ 7.28–7.33 (2H, m), 7.61 (1H, dd, J = 4.3 Hz, 8.2 Hz), 7.79–7.83 (2H, m), 8.48 (1H, dd, J = 2.1 Hz,8.2 Hz), 9.29 (1H, dd, J = 2.1 Hz, 4.3 Hz).

**2-(1H-benzo[*d*]imidazol-1-yl)-4-(4-fluorophenyl)pyrid[2,3-*d*]pyrimidine (S6)**

The preparation of **S6** was performed according to GP2A, using **S5** (301 mg, 1.16 mmol), benzimidazole (164 mg, 1.39 mmol), Cs_2_CO_3_ (566 mg, 1.74 mmol), XPhos (54.0 mg, 0.116 mmol), and Pd_2_(dba)_3_ (21.0 mg, 0.0232 mmol). Further purification was performed using column chromatography (hexane:AcOEt = 1:5) to afford **S6** (69.0 mg, 0.202 mmol, 17% yield) as a white solid.

^1^H-NMR (CDCl_3_) δ 7.35–7.50 (4H, m), 7.56 (1H, dd, J = 4.1 Hz, 8.2 Hz), 7.86 (1H, d, J = 7.3 Hz), 7.90–7.93 (2H, m), 8.50 (1H, dd, J = 1.8 Hz, 8.3 Hz), 8.90 (1H, d, J = 7.3 Hz), 9.28 (1H, dd, J = 1.8 Hz, 4.1 Hz), 9.35(1H,s).

**2-(1H-benzo[*d*]imidazol-1-yl)-4-(4-fluorophenyl)pyrid[2,3-*d*]pyrimidine hydrochloride (Compound 3)**

The preparation of Compound **3** was performed according to GP3, using **S6** (69.0 mg, 0.202 mmol), 4 N HCl/dioxane solution (0.5 mL), and MeOH (6 mL). Compound **3** (78.0 mg) was obtained as a white solid.

^1^H-NMR (DMSO-d_6_) δ 7.45 (1H, dd, J = 7.3 Hz, 8.0 Hz), 7.53–7.59 (3H, m), 7.77 (1H, dd, J = 4.4 Hz, 8.4 Hz), 7.85 (1H, d, J = 8.0 Hz), 8.08–8.12 (2H, m), 8.61 (1H, dd, J = 2.0 Hz, 8.4 Hz), 8.84 (1H, d, J = 8.0 Hz), 9.30 (1H, dd, J = 2.0Hz, 4.4 Hz), 9.44(1H,s).

**Synthesis of Compound 4**

**2,4-Dichloro-7-ethyl-7*H*-pyrrolo[2,3-*d*]pyrimidine (S7)**

A solution of 2,4-dichloro-7*H*-pyrrolo[2,3-*d*]pyrimidine (495 mg, 2.63 mmol) in DMF (13 mL) was cooled to 0 ºC. K_2_CO_3_ (545 mg, 3.95 mmol) and EtI (0.252 mL, 3.15 mmol) were added to this solution and stirred at room temperature for 30 min. After adding H_2_O (200 mL) to the reaction solution, the precipitated solid was filtered and dried to produce **S7** as a white solid (548 mg, 2.54 mmol, 96% yield).

^1^H-NMR (CDCl_3_) δ 1.50 (3H, t, J = 7.3 Hz), 4.31 (2H, q, J = 7.3 Hz), 6.62 (1H, d, J = 3.2 Hz), 7.26 (1H, d, J = 3.2 Hz).

**2-Chloro-7-ethyl-4-(4-fluorophenyl)-7*H*-pyrrolo[2,3-*d*]pyrimidine (S8)**

The preparation of **S8** was performed according to GP1B, using **S7** (84.8 mg, 0.392 mmol), 4-fluorophenylboronic acid (52.6 mg, 0.431 mmol), K_2_CO_3_ (81.2 mg, 0.588 mmol), and Pd(PPh_3_)_4_ (45.3 mg, 0.0392 mmol). Further purification was performed using column chromatography (hexane:AcOEt = 2:1) to afford **S8** (93.0 mg, 0.337 mmol, 86% yield) as a white solid.

^1^H-NMR (CDCl_3_ δ 1.52 (3H, t, J = 7.4 Hz), 4.35 (2H, q, J = 7.4 Hz), 6.79 (1H, d, J = 3.7 Hz), 7.20–7.27 (2H, m), 7.29 (1H, d, J = 3.7 Hz), 8.11–8.17 (2H, m).

**2-(1*H*-benzo[*d*]imidazol-1-yl)-7-ethyl-4-(4-fluorophenyl)-7*H*-pyrrolo[2,3-*d*]pyrimidine (S9)**

The preparation of **S9** was performed according to GP2A, using **S8** (87.3 mg, 0.316 mmol), benzimidazole (45.0 mg, 0.380 mmol), Cs_2_CO_3_ (155 mg, 0.474 mmol), XPhos (15.1 mg, 0.030 mmol), and Pd_2_(dba)_3_ (5.80 mg, 0.0063 mmol). Further purification was performed using column chromatography (hexane:AcOEt = 1:1) to afford **S9** (46.3 mg, 0.130 mmol, 41% yield) as a white solid.

^1^H-NMR (CDCl_3_) δ 1.62 (3H, t, J = 7.3 Hz), 4.45 (2H, q, J = 7.3 Hz), 6.84 (1H, d, J = 3.7 Hz), 7.28–7.35 (3H, m), 7.37–7.43 (1H, m), 7.44–7.50 (1H, m), 7.90 (1H, d, J = 7.3 Hz), 8.24–8.30 (2H, m), 8.78 (1H, d, J = 7.3 Hz), 9.29 (1H,s).

**2-(1*H*-benzo[*d*]imidazol-1-yl)-7-ethyl-4-(4-fluorophenyl)-7*H*-pyrrolo[2,3-*d*]pyrimidine hydrochloride (Compound 4)**

The preparation of Compound **4** was performed according to GP3, using **S9** (45.0 mg, 0.126 mmol), 4 N HCl/dioxane solution (0.4 mL), and MeOH (5 mL). Compound **4** (35.4 mg) was obtained as a white solid.

^1^H-NMR (CD_3_OD) δ 1.60 (3H, t, J = 7.4 Hz), 4.54 (2H, q, J = 7.4 Hz), 7.09 (1H, d, J = 3.9 Hz), 7.36–7.44 (2H, m), 7.71–7.76 (1H, m), 7.77 (1H, d, J = 3.9 Hz), 7.79–7.85 (1H, m), 7.95 (1H, d, J = 8.2 Hz), 8.39–8.45 (2H, m), 9.09 (1H, d, J = 8.2 Hz), 10.45(1H, s).

**Synthesis of Compound 5**

**2-Chloro-4-(furan-3-yl)-7-methyl-7*H*-pyrrolo[2,3-*d*]pyrimidine (S10)**

The preparation of **S10** was performed according to GP1A, using **S1** (150 mg, 0.742 mmol), 3-furylboronic acid (87.5 mg, 0.782 mmol), 1 N Na_2_CO_3_ aq. (1.40 mL, 1.40 mmol), and PdCl_2_(PPh_3_)_2_ (53.2 mg, 0.0758 mmol). Further purification was performed using column chromatography (hexane:AcOEt = 2:1) to afford **S10** (134 mg, 0.573 mmol, 77% yield) as a white solid.

^1^H-NMR (CDCl_3_) δ 3.87 (3H, s), 6.68 (1H, d, J = 3.8 Hz), 7.14 (1H, dd, J = 0.9 Hz, 1.8 Hz), 7.19 (1H, d, J = 3.8 Hz), 7.57 (1H, dd, J = 1.8 Hz, 1.8 Hz), 8.27–8.28 (1H, m).

**2-(1H-benzo[*d*]imidazol-1-yl)-4-(furan-3-yl)-7-methyl-7*H*-pyrrolo[2,3-*d*]pyrimidine (S11)**

The preparation of **S11** was performed according to GP2B, using **S10** (114 mg, 0.488 mmol), benzimidazole (69.1 mg, 0.585 mmol), K_3_PO_4_ (155 mg, 0.732 mmol), 2-(Di-tert-butylphosphino)-3,4,5,6-tetramethyl-2′,4′,6'-triisopropyl-1,1′-biphenyl (23.5 mg, 0.0488 mmol), and Pd_2_(dba)_3_ (8.90 mg, 0.00976 mmol). Further purification was performed using column chromatography (hexane:AcOEt = 1:1) to afford **S11** (21.1 mg, 0.0669 mmol, 14% yield) as a white solid.

^1^H-NMR (CDCl_3_) δ 3.83 (3H, s), 6.59 (1H, d, J = 3.6 Hz), 7.07 (1H, d, J = 3.6 Hz), 7.16 (1H, dd, J = 1.0 Hz, 1.7 Hz), 7.36 (1H, ddd, J = 1.3 Hz, 7.6 Hz, 7.6 Hz), 7.42 (1H, ddd, J = 1.3 Hz, 7.6 Hz, 7.6 Hz), 7.59 (1H, dd, J = 1.6 Hz, 1.6 Hz), 7.86 (1H, d, J = 7.6 Hz), 8.26 (1H, s), 8.68 (1H, dd, J = 1.0 Hz, 8.2 Hz), 9.15 (1H, s).

**2-(1H-benzo[*d*]imidazol-1-yl)-4-(furan-3-yl)-7-methyl-7*H*-pyrrolo[2,3-*d*]pyrimidine hydrochloride (Compound 5)**

The preparation of compound **5** was performed according to GP3, using **S11** (21.1 mg, 0.0669 mmol), 4 N HCl/dioxane solution (0.4 mL), and MeOH (2 mL). Compound **5** (20.1 mg) was obtained as a white solid.

^1^H-NMR (CD_3_OD) δ 3.93 (3H, s), 6.99 (1H, d, J = 3.6 Hz), 7.34 (1H, dd, J = 0.8 Hz, 2.0 Hz), 7.58 (1H, d, J = 3.6 Hz), 7.71–7.82 (3H, m), 7.90–7.93 (1H, m), 8.63–8.64 (1H, m), 9.03 (1H, d, J = 8.0 Hz), 10.41 (1H, s).

**Synthesis of Compound 6**

**2-Chloro-7-methyl-4-(thiophen-3-yl)-7*H*-pyrrolo[2,3-*d*]pyrimidine (S12)**

The preparation of **S12** was performed according to GP1A, using **S1** (150 mg, 0.742 mmol), 3-thiopheneboronic acid (99.5 mg, 0.778 mmol), 1 N Na_2_CO_3_ aq. (1.40 mL, 1.40 mmol), and PdCl_2_(PPh_3_)_2_ (55.6 mg, 0.0792 mmol). Further purification was performed using column chromatography (hexane:AcOEt = 2:1) to afford **S12** (129 mg, 0.517 mmol, 70% yield) as a white solid.

^1^H-NMR (CDCl_3_) δ 3.87 (3H, s), 6.78 (1H, d, J = 3.7 Hz), 7.20 (1H, d, J = 3.7 Hz), 7.46 (1H, dd, J = 2.8 Hz, 5.0 Hz), 7.87 (1H, dd, J = 1.0 Hz, 5.0 Hz), 8.21 (1H, dd, J = 1.0 Hz, 2.8 Hz).

**2-(1H-benzo[*d*]imidazol-1-yl)-7-methyl-4-(thiophen-3-yl)-7*H*-pyrrolo[2,3-*d*]pyrimidine (S13)**

The preparation of **S13** was performed according to GP2B, using **S12** (90.0 mg, 0.360 mmol), benzimidazole (51.4 mg, 0.435 mmol), K_3_PO_4_ (119 mg, 0.561 mmol), 2-(Di-tert-butylphosphino)-3,4,5,6-tetramethyl-2′,4′,6′-triisopropyl-1,1′-biphenyl (21.2 mg, 0.0441 mmol), and Pd_2_(dba)_3_ (12.2 mg, 0.0133 mmol). Further purification was performed using column chromatography (hexane:AcOEt = 1:1) to afford **S13** (59.3 mg, 0.179 mmol, 50% yield) as a white solid.

^1^H-NMR (CDCl_3_) δ 3.88 (3H, s), 6.76 (1H, d, J = 3.4 Hz), 7.14 (1H, d, J = 3.4 Hz), 7.37 (1H, ddd, J = 1.2 Hz, 7.6 Hz, 7.6 Hz), 7.41–7.46 (1H, m), 7.50 (1H, dd, J = 3.2 Hz, 5.0 Hz), 7.86 (1H, d, J = 8.0 Hz), 7.94 (1H, dd, J = 1.2 Hz, 5.0 Hz), 8.24 (1H, dd, J = 1.2 Hz, 3.2 Hz), 8.73 (1H, d, J = 8.0 Hz), 9.21 (1H, s).

**2-(1H-benzo[*d*]imidazol-1-yl)-7-methyl-4-(thiophen-3-yl)-7*H*-pyrrolo[2,3-*d*]pyrimidine hydrochloride (Compound 6)**

The preparation of Compound **6** was performed according to GP3, using **S13** (59.3 mg, 0.179 mmol), 4 N HCl/dioxane solution (0.4 mL), and MeOH (2 mL). Compound **6** (51.2 mg) was obtained as a white solid.

^1^H-NMR (CD_3_OD) δ 3.96 (3H, s), 7.06 (1H, d, J = 3.6 Hz), 7.58 (1H, d, J = 3.6 Hz), 7.64 (1H, dd, J = 3.2 Hz, 5.2 Hz), 7.70–7.81 (2H, m), 7.89–7.93 (1H, m), 8.04 (1H, dd, J = 1.2 Hz, 5.2 Hz), 8.55 (1H, dd, J = 1.6 Hz, 3.2 Hz), 9.00–9.03 (1H, m), 10.38 (1H, s).

**Synthesis of Compound 7**

**2-Chloro-7-methyl-4-(pyridin-4-yl)-7*H*-pyrrolo[2,3-*d*]pyrimidine (S14)**

The preparation of **S14** was performed according to GP1A, using **S1** (301 mg, 1.49 mmol), 4-pyridylboronic acid (201 mg, 1.63 mmol), 1 N Na_2_CO_3_ aq. (3.00 mL, 3.00 mmol), and PdCl_2_(PPh_3_)_2_ (105 mg, 0.149 mmol). Further purification was performed using column chromatography (hexane:AcOEt = 1:2) to afford **S14** (179 mg, 0.733 mmol, 49% yield) as a white solid.

^1^H-NMR (DMSO-d_6_) δ 3,81 (3H, s), 7.04 (1H, d, J = 3.7 Hz), 7.80 (1H, d, J = 3.7 Hz), 8.06 (2H, dd, J = 1.8 Hz, 4.1 Hz), 8.78 (2H, dd, J = 1.4 Hz, 4.1 Hz).

**2-(1*H*-benzo[*d*]imidazol-1-yl)-7-methyl-4-(pyridin-4-yl)-7H-pyrrolo[2,3-*d*]pyrimidine (S15)**

The preparation of **S15** was performed according to GP2B, using **S14** (179 mg, 0.773 mmol), benzimidazole (129 mg, 1.10 mmol), K_3_PO_4_ (337 mg, 1.46 mmol), 2-(Di-tert-butylphosphino)-3,4,5,6-tetramethyl-2′,4′,6'-triisopropyl-1,1′-biphenyl (35.2 mg, 0.0733 mmol), and Pd_2_(dba)_3_ (67.1 mg, 0.0733 mmol). Further purification was performed using column chromatography (hexane:AcOEt = 1:4) to afford **S15** (230 mg, 0.704 mmol, 96% yield) as a white solid.

^1^H-NMR (DMSO-d_6_) δ 4.03 (3H, s), 7.13 (1H, d, J = 3.7 Hz), 7.39 (1H, dd, J = 7.3 Hz, 7.8 Hz), 7.50 (1H, dd, J = 7.8 Hz, 8.2 Hz), 7.81 (1H, d, J = 7.3 Hz), 7.83 (1H, d, J = 3.7 Hz), 8.31 (2H, dd, J = 1.8 Hz, 4.6 Hz), 8.77 (1H, d, J = 8.2 Hz), 8.88 (2H, dd, J = 1.4 Hz, 4.6 Hz), 9.41 (1H, s).

**2-(1*H*-benzo[*d*]imidazol-1-yl)-7-methyl-4-(pyridin-4-yl)-7H-pyrrolo[2,3-*d*]pyrimidine hydrochloride (Compound 7)**

The preparation of Compound **7** was performed according to GP3, using **S15** (230 mg, 0.704 mmol), 4 N HCl/dioxane solution (0.8 mL), and MeOH (5 mL). Compound **7** (231 mg) was obtained as a white solid.

^1^H-NMR (CD_3_OD) δ 4.12 (3H, s), 7.29 (1H, d, J = 3.7 Hz), 7.77 (1H, ddd, J = 0.9 Hz, 7.3 Hz, 8.2 Hz), 7.85 (1H, ddd, J = 0.9 Hz, 7.3 Hz, 8.2 Hz), 7.96 (1H, d, J = 3.7 Hz), 7.97 (1H, d, J = 8.2 Hz), 9.04 (2H, d, J = 6.9 Hz), 9.12 (1H, d, J = 8.2 Hz), 9.14 (2H, d, J = 6.9 Hz), 10.66 (1H, s).

**Synthesis of Compound 8**

**2-Chloro-7-methyl-4-(pyridin-3-yl)-7*H*-pyrrolo[2,3-*d*]pyrimidine (S16)**

The preparation of **S16** was performed according to GP1A, using **S1** (320 mg, 1.58 mmol), 3-pyridylboronic acid (212 mg, 1.72 mmol), 1 N Na_2_CO_3_ aq. (3.00 mL, 3.00 mmol), and PdCl_2_(PPh_3_)_2_ (111 mg, 0.158 mmol). Further purification was performed using column chromatography (hexane:AcOEt = 1:2) to afford **S16** (254 mg, 1.04 mmol, 66% yield) as a white solid.

**2-(1*H*-benzo[*d*]imidazol-1-yl)-7-methyl-4-(pyridin-3-yl)-7*H*-pyrrolo[2,3-*d*]pyrimidine (S17)**

The preparation of **S17** was performed according to GP2B, using **S16** (254 mg, 1.04 mmol), benzimidazole (183 mg, 1.55 mmol), K_3_PO_4_ (476 mg, 2.07 mmol), 2-(Di-tert-butylphosphino)-3,4,5,6-tetramethyl-2′,4′,6'-triisopropyl-1,1′-biphenyl (50.5 mg, 0.105 mmol), and Pd_2_(dba)_3_ (96.2 mg, 0.105 mmol). Further purification was performed using column chromatography (hexane:AcOEt = 1:4) to afford **S17** (261 mg, 0.800 mmol, 77% yield) as a white solid.

^1^H-NMR (DMSO-d_6_) δ 3.94 (3H, s), 7.06 (1H, d, J = 3.7 Hz), 7.34 (1H, dd, J = 7.8 Hz, 8.2 Hz), 7.45 (1H, dd, J = 8.2 Hz, 8.2 Hz), 7.66 (1H, dd, J = 5.0 Hz, 8.2 Hz), 7.74 (1H, d, J = 3.7 Hz), 7.77 (1H, d, J = 7.8 Hz), 8.71 (1H, ddd, J = 1.8 Hz, 1.8 Hz, 8.2 Hz), 8.73 (1H, d, J = 8.2 Hz), 8.78 (1H, dd, J = 1.8 Hz, 5.0 Hz), 9.38 (1H, s), 9.48 (1H, d, J = 1.8 Hz).

**2-(1*H*-benzo[*d*]imidazol-1-yl)-7-methyl-4-(pyridin-3-yl)-7*H*-pyrrolo[2,3-*d*]pyrimidine hydrochloride (Compound 8)**

The preparation of compound **8** was performed according to GP3, using **S17** (261 mg, 0.800 mmol), 4 N HCl/dioxane solution (1 mL), and MeOH (6 mL). Compound **8** (280 mg) was obtained as a white solid.

^1^H-NMR (CD_3_OD) δ 3.24 (3H, s), 7.26 (1H, d, J = 3.7 Hz), 7.77 (1H, dd, J = 7.3 Hz, 7.8 Hz), 7.84 (1H, dd, J = 7.8 Hz, 8.2 Hz), 7.89 (1H, d, J = 3.7 Hz), 7.97 (1H, d, J = 8.2 Hz), 8.34 (1H, dd, J = 6.0 Hz, 8.2 Hz), 9.07 (1H, dd, J = 1.8 Hz, 6.0 Hz), 9.11 (1H, d, J = 7.3 Hz), 9.56 (1H, ddd, J = 1.8 Hz, 1.8 Hz, 8.2 Hz), 9.86 (1H, d, J = 1.8 Hz), 10.67 (1H, s).

**Synthesis of Compound 9**

**4-(Benzyloxy)-2-chloro-7-methyl-7*H*-pyrrolo[2,3-*d*]pyrimidine (S18)**

A solution of **S1** (7.24 g, 35.8 mmol) in DMF (300 mL) was cooled to 0 ºC. NaH (2.15 g, 53.7 mmol), and BnOH (4.47 mL, 43.0 mmol) were added to this solution and stirred for 1 h. After adding water to the reaction mixture, it was extracted with diethyl ether. The organic layer was dried with anhydrous sodium sulfate, filtered, and the solvent was removed under reduced pressure. The residue was purified using silica gel column chromatography (hexane:AcOEt = 2:1) to afford **S18** (9.42 g, 34.4 mmol, 96% yield) as a white solid.

^1^H-NMR (CDCl_3_) δ 3.81 (3H, s), 5.56 (2H, s), 6.51 (1H, d, J = 3.2 Hz), 6.95 (1H, d, J = 3.2 Hz), 7.34–7.41 (3H, m), 7.49–7.51 (2H, m).

**2-(1H-benzo[*d*]imidazol-1-yl)-4-(benzyloxy)-7-methyl-7*H*-pyrrolo[2,3-*d*]pyrimidine (S19)**

The preparation of **S19** was performed according to GP2C, using **S18** (1.56 g, 5.69 mmol), benzimidazole (739 mg, 6.26 mmol), Cs_2_CO_3_ (2.40 g, 7.40 mmol), XPhos (271 mg, 0.569 mmol), and Pd_2_(dba)_3_ (260 mg, 0.284 mmol). Further purification was performed using column chromatography (hexane:AcOEt = 1:2) to afford **S19** (822 mg, 2.31 mmol, 41% yield) as a white solid.

^1^H-NMR (CDCl_3_) δ 3.86 (3H, s), 5.74 (2H, s), 6.57 (1H, d, J = 3.2 Hz), 7.30–7.42 (6H, m), 7.53–7.56 (2H, m), 7.75 (1H, d, J = 7.8 Hz), 8.63 (1H, d, J = 7.8 Hz), 9.22 (1H, s).

**2-(1*H*-benzo[*d*]imidazol-1-yl)-7-methyl-7*H*-pyrrolo[2,3-*d*]pyrimidin-4-ol (S20)**

Chloroform (15 mL) and methanol (15 mL) solutions of **S19** (790 mg, 2.20 mmol) were added to palladium-activated carbon (Pd 10%) (473 mg, 0.440 mmol) and stirred for 4 h under a hydrogen atmosphere. The reaction solution was filtered through celite, and the solvent was removed under reduced pressure. The residue was suspended in n-hexane and ethyl acetate, filtered, dried, and **S20** (581 mg, 2.19 mmol, 99% yield) was obtained.

^1^H-NMR (DMSO-d_6_) δ 3.83 (3H, s), 4.01 (1H, brs), 6.58 (1H, d, J = 3.2 Hz), 7.29 (1H, brs), 7.42 (1H, dd, J = 7.2 Hz, 8.0 Hz), 7.49 (1H, dd, J = 7.2 Hz, 8.0 Hz), 7.80 (1H, d, J = 8.4 Hz), 8.62 (1H, brs), 9.20 (1H, brs).

**2-(1*H*-benzo[*d*]imidazol-1-yl)-7-methyl-7*H*-pyrrolo[2,3-*d*]pyrimidin-4-yl 4-methylbenzenesulfonic acid (S21)**

A solution of **S20** (323 mg, 1.22 mmol) in DMF (10 mL) was prepared by adding triethylamine (0.255 mL, 1.83 mmol) and *p*-TsCl (279 mg, 1.46 mmol), and the mixture was stirred at room temperature for 1 h. The reaction mixture was poured into ice water, and the precipitated solid was filtered off, dried, and **S21** (376 mg, 0.896 mmol, 73% yield) was obtained.

^1^H-NMR (DMSO-d_6_) δ 2.45 (3H, s), 3.94 (3H, s), 6.67 (1H, d, J = 3.7 Hz), 7.35–7.41 (2H, m), 7.55 (2H, d, J = 7.8 Hz), 7.71 (1H, d, J = 3.7 Hz), 7.75–7.80 (1H, m), 8.03 (2H, d, J = 8.2 Hz), 8.32–8.37 (1H, m), 8.77 (1H, s).

***tert*-Butyl 6-(2-(1*H*-benzo[*d*]imidazol-1-yl)-7-methyl-7*H*-pyrrolo[2,3-*d*]pyrimidin-4-yl)-3,4-dihydroisoquinolin-2(1*H*)-carboxylate (S22)**

The preparation of **S22** was performed according to GP1C, using **S21** (160 mg, 0.381 mmol), *tert*-butyl 3-(tetramethyl-1,3,2-dioxaborolan-2-yl)-6,8-dihydro-5*H*-1,7-naphthyridine-7-carboxylate (200 mg, 0.557 mmol), K_2_CO_3_ (70.0 mg, 0.506 mmol), and Pd(PPh_3_)_4_ (40.0 mg, 0.0346 mmol). Further purification was performed using column chromatography (hexane:AcOEt = 1:3) to afford **S22** (160 mg, 0.333 mmol, 87% yield) as a white solid.

^1^H-NMR (DMSO-d_6_) δ 1.46 (9H, s), 3.13–3.16 (2H, m), 3.33–3.63 (2H, m), 3.97 (3H, s), 4.70–4.72 (2H, m), 7.08 (1H, d, J = 3.7 Hz), 7.36–7.66 (3H, m), 7.71 (1H, d, J = 3.7 Hz), 8.14–8.17 (2H, m), 8.78 (1H, d, J = 8.2 Hz), 9.41 (1H, s).

**6-(2-(1*H*-benzo[*d*]imidazol-1-yl)-7-methyl-7*H*-pyrrolo[2,3-*d*]pyrimidin-4-yl)-1,2,3,4-tetrahydroisoquinoline hydrochloride (Compound 9)**

HCl/dioxane solution (4 N; 4 mL) was added to **S22** (160 mg, 0.333 mmol) and stirred at room temperature for 2 h. The precipitated solid was filtered, washed with acetone, dried, and compound **9** (133 mg) was obtained.

^1^H-NMR (DMSO-d_6_) δ 3.21–3.25 (2H, m), 3.53–3.57 (2H, m), 3.99 (3H, s), 4.42–4.44 (2H, m), 7.05 (1H, d, J = 3.6 Hz), 7.35–7.39 (1H, m), 7.46–7.48 (2H, m), 7.73 (1H, d, J = 3.8 Hz), 7.79 (1H, d, J = 8.0 Hz), 8.19–8.21 (2H, m), 8.76 (1H, d, J = 7.9 Hz), 9.44 (1H, s).

**Synthesis of Compound 10**

***tert*-Butyl 5-(2-(1*H*-benzo[*d*]imidazol-1-yl)-7-methyl-7*H*-pyrrolo[2,3-*d*]pyrimidin-4-yl)-2,3-dihydro-1H-pyrrolo[2,3-*b*]pyridin-1-carboxylate (S23)**

The preparation of **S23** was performed according to GP1C, using **S21** (156 mg, 0.371 mmol), *tert*-butyl 5-(4,4,5,5-tetramethyl-1,3,2-dioxaborolan-2-yl)-2,3-dihydro-1*H*-pyrrolo[2,3-*b*]pyridine-1-carboxylate (193 mg, 0.557 mmol), K_2_CO_3_ (66.6 mg, 0.482 mmol), and Pd(PPh_3_)_4_ (42.9 mg, 0.0371 mmol). Further purification was performed using column chromatography (hexane:AcOEt = 1:2) to afford **S23** (170 mg, 0.364 mmol, 98% yield) as a white solid.

^1^H-NMR(CDCl_3_) δ 1.61 (9H, s), 3.24 (2H, t, J = 8.6 Hz), 3.98 (3H, s), 4.14 (2H, t, J = 8.8 Hz), 6.84 (1H, d, J = 3.6 Hz), 7.26 (1H, d, J = 3.2 Hz), 7.34–7.42 (1H, m), 7.42–7.50 (1H, m), 7.88 (1H, d, J = 7.6 Hz), 8.30–8.37 (1H, m), 8.78 (1H, d, J = 8.0 Hz), 9.13 (1H, d, J = 1.6 Hz), 9.26 (1H, s).

**2-(1*H*-benzo[*d*]imidazol-1-yl)-4-(2,3-dihydro-1*H*-pyrrolo[2,3-b]pyridin-5-yl)-7-methyl-7H-pyrrolo[2,3-*d*]pyrimidine hydrochloride (Compound 10)**

**S23** (170 mg, 0.382 mmol) was dissolved in ethanol (8 mL), and 4 N HCl/dioxane solution (4 mL) was added. The mixture was refluxed for 1 h. After cooling, the solvent was removed under reduced pressure. Ethyl acetate and diethyl ether were added to the residue, and the resulting solid was filtered, dried, and compound **10** (153 mg) was obtained.

^1^H-NMR (CD_3_OD) δ 3.43 (2H, t, J = 8.0 Hz), 4.03–4.11 (5H, m), 7.12 (1H, d, J = 4.4 Hz), 7.71–7.79 (2H, m), 7.83 (1H, dd, J = 7.6 Hz, 8.0 Hz), 7.96 (1H, d, J = 7.6 Hz), 8.56–8.65 (2H, m), 9.03–9.12 (1H, m), 10.48–10.61 (1H, m).

**Synthesis of Compound 11**

***tert*-Butyl 6-(2-chloro-7-methyl-7*H*-pyrrolo[2,3-*d*]pyrimidin-4-yl)-3,4-dihydroisoquinoline-2(1*H*) carboxylic acid (S24)**

The preparation of **S24** was performed according to GP1A, using **S1** (100 mg, 0.495 mmol), *tert*-butyl 3-(tetramethyl-1,3,2-dioxaborolan-2-yl)-6,8-dihydro-5*H*-1,7-naphthyridine-7-carboxylate (190 mg, 0.495 mmol), 1 N Na_2_CO_3_ aq. (1 mL, 1 mmol), and PdCl_2_(PPh_3_)_2_ (37.2 mg, 0.0530 mmol). Further purification was performed using column chromatography (hexane:AcOEt = 1:1) to afford **S24** (120 mg, 0.301 mmol, 61% yield) as a white solid.

^1^H-NMR (CDCl_3_) δ 1.51 (9H, s), 2.96 (2H, t, J = 5.5 Hz), 3.70 (2H, t, J = 5.5 Hz), 3.89 (3H, s), 4.66 (2H, s), 6.80 (1H, d, J = 3.7 Hz), 7.21 (1H, d, J = 3.7 Hz), 7.26–7.30 (1H, m), 7.90–7.94 (2H, m).

***tert*-Butyl 6-(7-methyl-2-(4-methyl-1*H*-benzo[d]imidazol-1-yl)-7*H*-pyrrolo[2,3-*d*]pyrimidin-4-yl)-3,4-dihydroisoquinoline-2(1*H*) carboxylic acid (S25)**

The preparation of **S25** was performed according to GP2A, using **S24** (67.4 mg, 0.257 mmol), 4-methylbenzimidazole (128 mg, 0.332 mmol), Cs_2_CO_3_ (162 mg, 0.497 mmol), XPhos (17.9 mg, 0.0375 mmol), and Pd_2_(dba)_3_ (17.3 mg, 0.0189 mmol). Further purification was performed using column chromatography (NH, hexane:AcOEt = 1:1) to afford **S25** (72.1 mg, 0.146 mmol, 45% yield) as a white solid.

^1^H-NMR (CDCl_3_) δ 1.53 (9H, s), 2.75 (3H, s), 3.01 (2H, t, J = 5.5 Hz), 3.75 (2H, t, J = 5.5 Hz), 3.99 (3H, s), 4.71 (2H, s), 6.84 (1H, d, J = 3.7 Hz), 7.19 (1H, d, J = 7.1 Hz), 7.23 (1H, d, J = 3.7 Hz), 7.31–7.38 (2H, m), 8.02 (1H, s), 8.06 (1H, d, J = 8.2 Hz), 8.64 (1H, d, J = 7.8 Hz), 9.26 (1H, s).

**6-(7-Methyl-2-(4-methyl-1*H*-benzo[*d*]imidazol-1-yl)-7*H*-pyrrolo[2,3-d]pyrimidin-4-yl)-1,2,3,4-tetrahydroisoquinoline hydrochloride (Compound 11)**

**S25** (70.0 mg, 0.142 mmol) was dissolved in methanol (4 mL), and 4 N HCl/dioxane solution (0.8 mL) was added. The mixture was refluxed for 1 h. After cooling, the solvent was removed under reduced pressure. Ethyl acetate and diethyl ether were added to the residue, and the resulting solid was filtered, dried, and compound **11** (49.5 mg) was obtained.

^1^H-NMR (DMSO-d_6_) δ 2.65 (3H, s), 3.23 (2H, t, J = 6.2 Hz), 3.43–3.50 (2H, m), 3.99 (3H, s), 4.38–4.43 (2H, m), 7.09 (1H, d, J = 3.7 Hz), 7.27 (1H, d, J = 7.3 Hz), 7.43 (1H, dd, J = 7.8 Hz, 7.8 Hz), 7.51 (1H, d, J = 7.8 Hz), 7.79 (1H, d, J = 3.7 Hz), 8.20-8.25 (2H, m), 8.64 (1H, d, J = 8.2 Hz), 9.50 (1H, s), 9.63 (1H, s).

**Synthesis of ZSTK3744 (Compound 12)**

***tert*-Butyl 5-(2-chloro-7-methyl-7*H*-pyrrolo[2,3-*d*]pyrimidin-4-yl)-2,3-dihydro-1*H*-pyrrolo[2,3-*b*]pyridine-1-carboxylate (S26)**

The preparation of **S26** was performed according to GP1B, using **S1** (3.75 g, 18.5 mmol), *tert*-butyl 5-(4,4,5,5-tetramethyl-1,3,2-dioxaborolan-2-yl)-2,3-dihydro-1*H*-pyrrolo[2,3-*b*]pyridine-1-carboxylate (6.56 g, 18.9 mmol), K_2_CO_3_ ((3.32 g, 24.1 mmol), and Pd(PPh_3_)_4_ (303 mg, 0.263 mmol). Further purification was performed using column chromatography (hexane:AcOEt = 2:1) to afford **S26** (5.73 g, 14.9 mmol, 80% yield) as a white solid.

^1^H-NMR(DMSO-d_6_) δ 1.52 (9H, s), 3.15 (2H, t, J = 8.7 Hz), 3.82 (3H, s), 4.02 (2H, t, J = 8.7 Hz), 7.05 (1H, d, J = 3.7 Hz), 7.73 (1H, d, J = 3.7 Hz), 8.27 (1H, d, J = 1.8 Hz), 8.87 (1H, d, J = 1.8 Hz).

***tert*-Butyl 5-(7-methyl-2-(4-methyl-1*H*-benzo[*d*]imidazol-1-yl)-7*H*-pyrrolo[2,3-*d*]pyrimidin-4-yl)-2,3-dihydro-1*H*-pyrrolo[2,3-*b*]pyridine-1-carboxylate (S27)**

The preparation of **S27** was performed according to GP2A, using **S26** (5.73 g, 14.8 mmol), 4-methylbenzimidazole (2.55 g, 19.3 mmol), Cs_2_CO_3_ (6.29 g, 19.3 mmol), XPhos (1.41 g, 2.97 mmol), and Pd_2_(dba)_3_ (1.35 g, 1.48 mmol). Further purification was performed using column chromatography (NH, hexane:AcOEt = 1:1) to afford **S27** (4.80 g, 9.97 mmol, 67% yield) as a white solid.

^1^H-NMR(CDCl_3_) δ 1.61 (9H, s), 2.75 (3H, s), 3.24 (2H, t, J = 8.2 Hz), 3.98 (3H, s), 4.14 (2H, t, J = 8.2 Hz), 6.48 (1H, d, J = 3.7 Hz), 7.19 (1H, d, J = 7.3 Hz), 7.24 (1H, d, J = 3.7 Hz), 7.35 (1H, dd, J = 7.3 Hz, 8.2 Hz), 8.33 (1H, d, J = 2.3 Hz), 8.61 (1H, d, J = 8.2 Hz), 9.14 (1H, d, J = 2.3 Hz), 9.23 (1H, s).

**4-(2,3-dihydro-1*H*-pyrrolo[2,3-*b*]pyridin-5-yl)-7-methyl-2-(4-methyl-1*H*-benzo[*d]*imidazol-1-yl)-7*H*-pyrrolo[2,3-*d*]pyrimidine hydrochloride (ZSTK3744)**

**S27** (135 mg, 0.280 mmol) was dissolved in ethanol (8 mL), and 4 N HCl/dioxane solution (4 mL) was added. The mixture was refluxed for 1 h. After cooling, the solvent was removed under reduced pressure. Ethyl acetate and diethyl ether were added to the residue, and the resulting solid was filtered, dried, and ZSTK3744 (115 mg) was obtained.

^1^H-NMR (DMSO-d_6_) δ 2.61 (3H, s), 3,27 (2H, t, J = 8.2 Hz), 3.88 (2H, t, J = 8.2 Hz), 3.93 (3H, s), 7.05 (1H, d, J = 3.7 Hz), 7.20 (1H, d, J = 7.3 Hz), 7.36 (1H, dd, J = 7.3 Hz, 8.2 Hz), 7.74 (1H, d, J = 3.7 Hz), 8.52 (1H, d, J = 1.4 Hz), 8.54 (1H, d, J = 8.2 Hz), 8.57 (1H, d, J = 1.4 Hz), 9.03 (1H, s), 9.52 (1H, s). ^13^C-NMR (CD_3_OD) δ 16.7, 27.1, 32.1, 47.7, 101.8, 115.4, 116.0, 123.7, 127.2, 129.0, 129.1, 131.0, 133.5, 133.6, 134.9, 134.9, 135.4, 142.2, 149.7, 153.7, 154.2, 158.6. m/z: (APCI) [M + H]^+^ = 381.9.

**List of abbreviations**

DMF, dimethylformamide; DMSO, dimethyl sulfoxide; DME, 1,2-dimethoxyethane; MeOH, methanol; EtOH, ethanol; AcOEt, ethyl acetate; Boc, *tert*-butoxycarbonyl; MeI, iodomethane; Na_2_CO_3_, sodium carbonate; K_2_CO_3_, potassium carbonate; XPhos, 2-dicyclohexylphosphino-2',4',6'-triisopropylbiphenyl; PdCl_2_(PPh_3_)_2_, bis(triphenylphosphine)palladium(II) dichloride; Pd_2_(dba)_3_, tris(dibenzylideneacetone)dipalladium(0); Pd(PPh_3_)_4_, tetrakis(triphenylphosphine)palladium(0)
