# Supplementary material for: ZSTK3744, a Novel Aryl Hydrocarbon Receptor Agonist, Exhibits Efficacy against Chemotherapy-Resistant Triple-Negative Breast Cancer
Source: Cancer Res Commun. 2026 Feb 27;6(2):421–36. doi: 10.1158/2767-9764.CRC-25-0119 (PMC13148475; doi:10.1158/2767-9764.CRC-25-0119)
Supplement: Supplementary Figure S2 — Investigation of the effects of ABCB1 on chemotherapy resistance [file crc-25-0119_supplementary_figure_s2_suppsf2.docx]

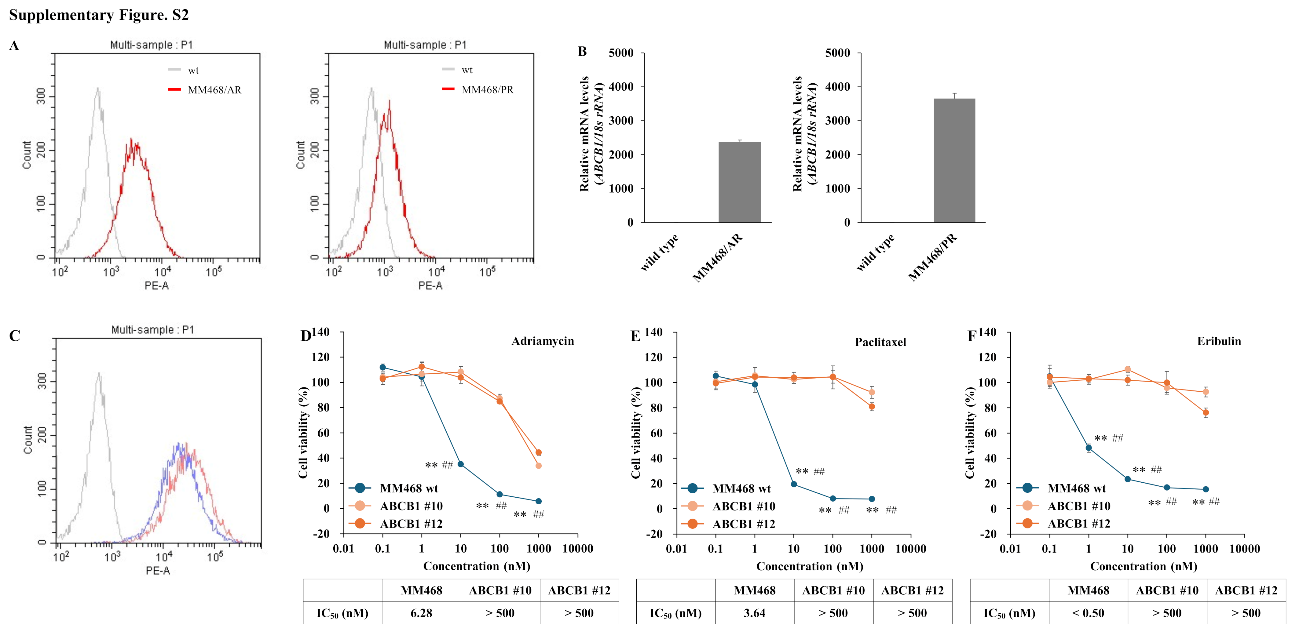


**Supplementary Fig. S2. Investigation of the effects of ABCB1 on chemotherapy resistance**

(A) ABCB1 expression levels on the cell membrane of MM468/AR and MM468/PR cells were analyzed using flow cytometry. (B) ABCB1 mRNA expression levels in MM468/AR and MM468/PR cells were analyzed using real-time PCR. (C) ABCB1 expression levels on the cell membrane of parental MM468 (gray) and ABCB1-overexpressed MM468 cells clone #10 (blue) and #12 (red) were analyzed using flow cytometry. Parental MM468 and ABCB1-overexpressed cells were treated with adriamycin (D), paclitaxel (E), and eribulin (F) at the indicated concentrations for 72 h. Cell viability was measured using the Cell Counting Kit-8 assay (mean ± SD, n = 4).
