# Supplementary material for: ZSTK3744, a Novel Aryl Hydrocarbon Receptor Agonist, Exhibits Efficacy against Chemotherapy-Resistant Triple-Negative Breast Cancer
Source: Cancer Res Commun. 2026 Feb 27;6(2):421–36. doi: 10.1158/2767-9764.CRC-25-0119 (PMC13148475; doi:10.1158/2767-9764.CRC-25-0119)
Supplement: Supplementary Figure S3 — Evaluation of cell growth-inhibitory effects of standard chemotherapy agents on resistant and ABCB1-overexpressing cells [file crc-25-0119_supplementary_figure_s3_suppsf3.docx]

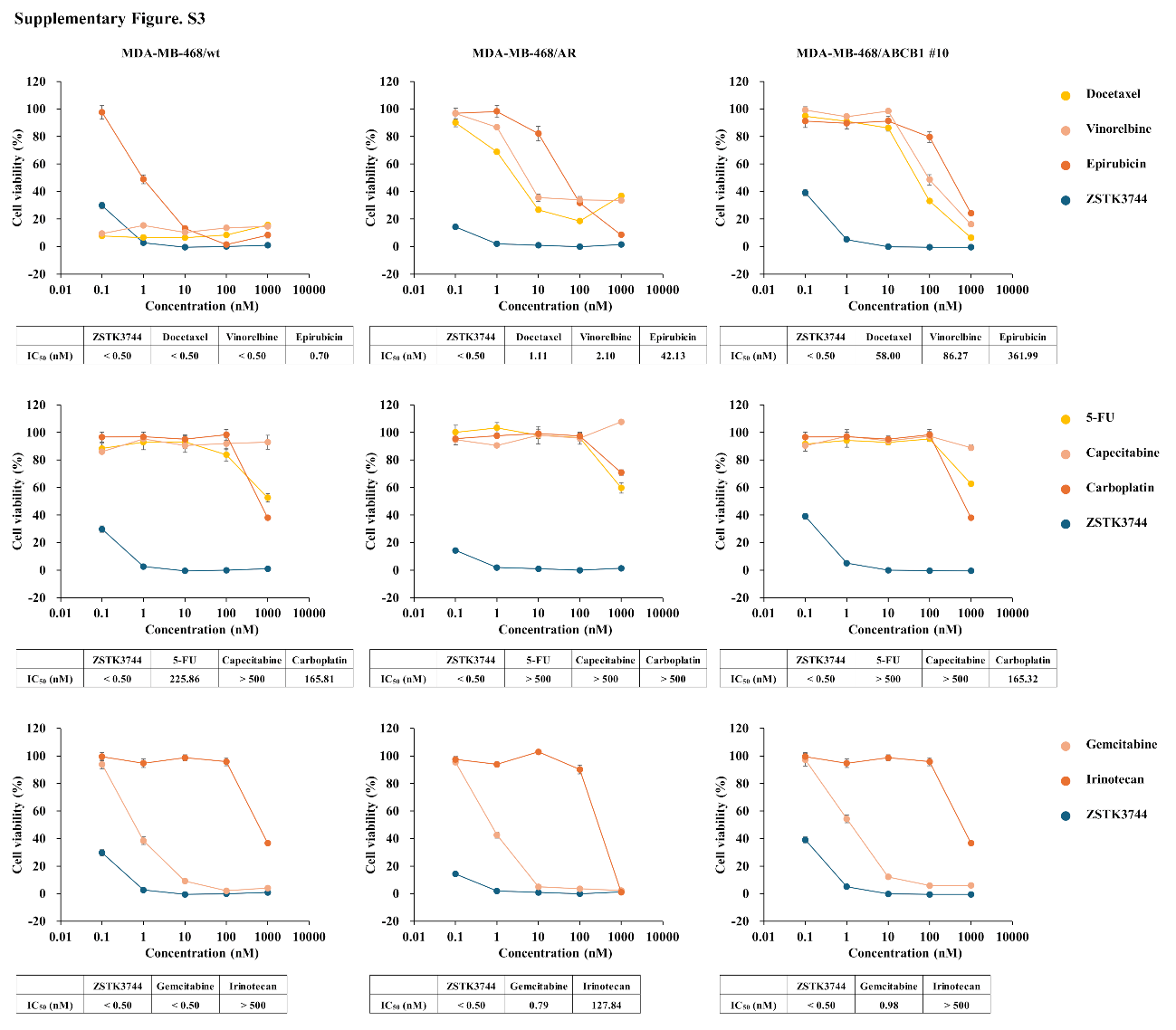


**Supplementary Fig. S3. Evaluation of cell growth-inhibitory effects of standard chemotherapy agents on resistant and ABCB1-overexpressing cells**

Parental MM468 (left), MM468/AR (middle), and MM468/ABCB1 #10 (right) cells were treated with ZSTK3744, docetaxel, vinorelbine, epirubicin (upper panel), 5-fluorouracil (5-FU), capecitabine, carboplatin (middle panel), gemcitabine, and irinotecan (bottom panel) at the indicated concentrations for 72 h. Cell viability was assessed using the Cell Counting Kit-8 assay (mean ± SD, n = 4). Docetaxel (047-31281, FUJIFILM Wako Pure Chemical Corp., Osaka, Japan), vinorelbine (057-402734, Kyowa Kirin, Tokyo, Japan), epirubicin (E9406, Merck), 5-FU (Kyowa Kirin), capecitabine (034-23441, FUJIFILM Wako Pure Chemical Corp.), carboplatin (033-25231, FUJIFILM Wako Pure Chemical Corp.), gemicitabine (G6423-50MG, Merck), and irinotecan (091-06651, FUJIFILM Wako Pure Chemical Corp.) were used as standard chemotherapy agents for comparison.
