# Supplementary material for: ZSTK3744, a Novel Aryl Hydrocarbon Receptor Agonist, Exhibits Efficacy against Chemotherapy-Resistant Triple-Negative Breast Cancer
Source: Cancer Res Commun. 2026 Feb 27;6(2):421–36. doi: 10.1158/2767-9764.CRC-25-0119 (PMC13148475; doi:10.1158/2767-9764.CRC-25-0119)
Supplement: Supplementary Figure S4 — Evaluation of PI3K enzyme activity [file crc-25-0119_supplementary_figure_s4_suppsf4.docx]

**
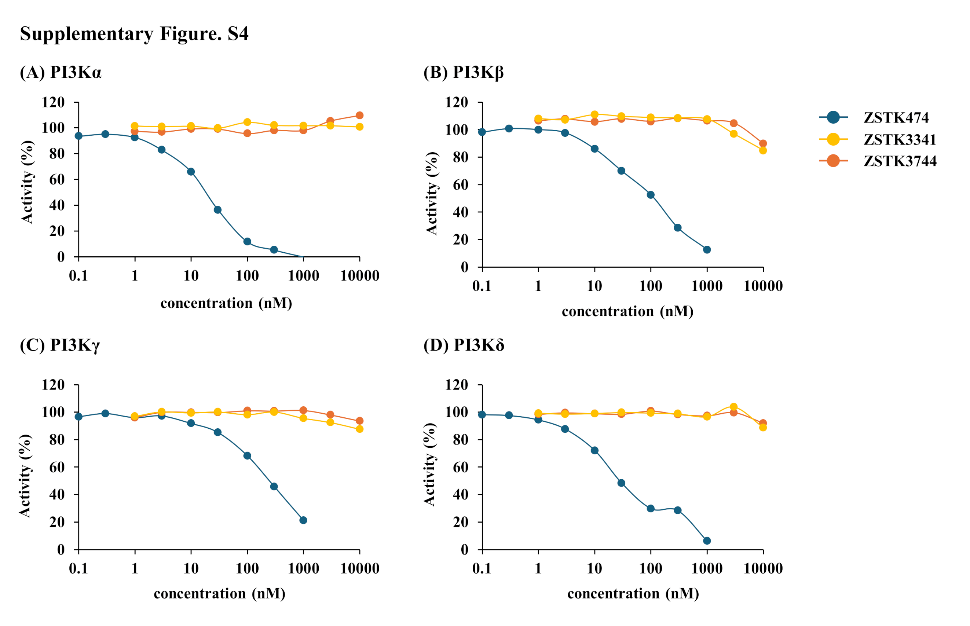
**

**Supplementary Fig. S4. Evaluation of PI3K enzyme activity**

The kinase activities of PI3K α (A), β (B), γ (C), and δ (D) were determined using the PI3K HTRF assay kit according to the manufacturer’s instructions. ZSTK474 was treated at concentrations of 0.1, 0.3, 1, 3, 10, 30, 100, 300, and 1,000 nM, while ZSTK3341 and ZSTK3744 were treated at 1, 3, 10, 30, 100, 300, 1,000, 3,000, and 10,000 nM. Fluorescence was measured at 665 and 620 nm using a Wallac EnVision 2102 Multilabel Reader. Enzyme activity was expressed as a percentage of the non-treated control (mean, n = 3).
