# Supplementary material for: ZSTK3744, a Novel Aryl Hydrocarbon Receptor Agonist, Exhibits Efficacy against Chemotherapy-Resistant Triple-Negative Breast Cancer
Source: Cancer Res Commun. 2026 Feb 27;6(2):421–36. doi: 10.1158/2767-9764.CRC-25-0119 (PMC13148475; doi:10.1158/2767-9764.CRC-25-0119)
Supplement: Supplementary Figure S5 — Analysis of apoptotic cells [file crc-25-0119_supplementary_figure_s5_suppsf5.docx]

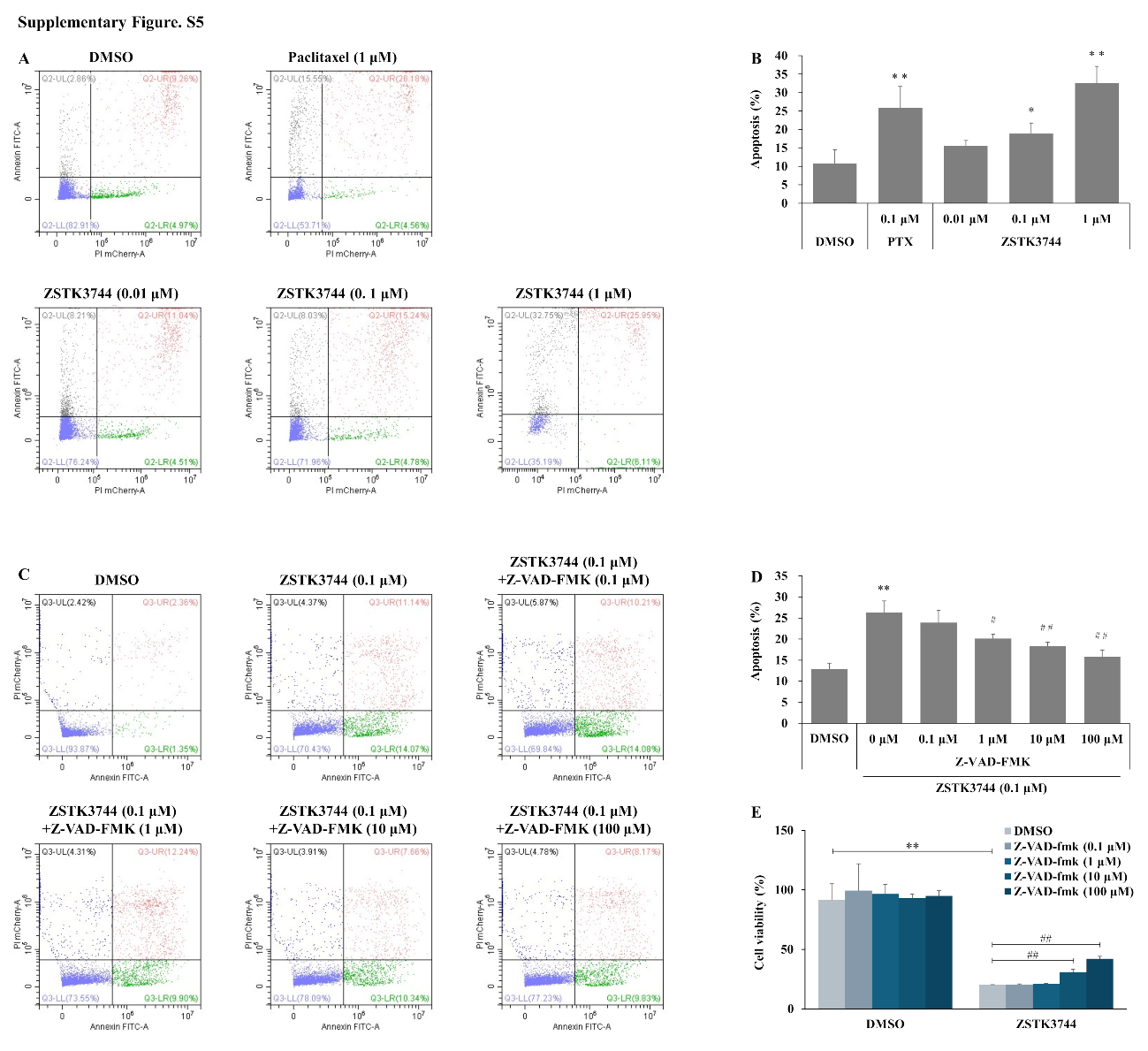


**Supplementary Fig. S5. Analysis of apoptotic cells**

(A and B) MM468 cells were treated with paclitaxel (0.1 μM) and ZSTK3744 (0.01, 0.1, and 1 μM) for 24 h. Apoptotic cells were stained with annexin V and propidium iodide (PI) and analyzed using flow cytometry. The percentage of apoptotic cells was calculated as the sum of early (annexin V-positive cells) and late apoptosis (annexin V/PI double-positive cells). Statistical analysis was performed with one-way analysis of variance (ANOVA), followed by Dunnett’s test (**p < 0.01, *p < 0.05 versus DMSO-treated cells). (C and D) MM468 cells were pretreated with Z-VAD-FMK, pan-caspase inhibitor (0.1, 1, 10, and 100 μM), 30 min before ZSTK3744 (0.1 μM) treatment, and then incubated for 24 h. Apoptotic cells were analyzed using flow cytometry. Statistical analysis was performed with one-way ANOVA, followed by Dunnett’s test (***p < 0.005 versus DMSO-treated cells and ^##^p < 0.01, ^#^p < 0.05 versus ZSTK3744-treated cells). (E) MM468 cells were treated with ZSTK3744 alone (0.1 μM) or in combination with the Z-VAD-FMK (0.1, 1, 10, and 100 μM) for 72 h, and cell viability was assessed using the Cell Counting Kit-8 assay (mean ± SD of quadruplicate experiments). Statistical analysis was performed with one-way ANOVA, followed by Dunnett’s test (**p < 0.01 versus DMSO-treated cells and ^##^p < 0.01 versus ZSTK3744-treated cells).
