# Supplementary material for: ZSTK3744, a Novel Aryl Hydrocarbon Receptor Agonist, Exhibits Efficacy against Chemotherapy-Resistant Triple-Negative Breast Cancer
Source: Cancer Res Commun. 2026 Feb 27;6(2):421–36. doi: 10.1158/2767-9764.CRC-25-0119 (PMC13148475; doi:10.1158/2767-9764.CRC-25-0119)
Supplement: Supplementary Figure S6 — Evaluation of biomarkers for ZSTK3744 [file crc-25-0119_supplementary_figure_s6_suppsf6.docx]

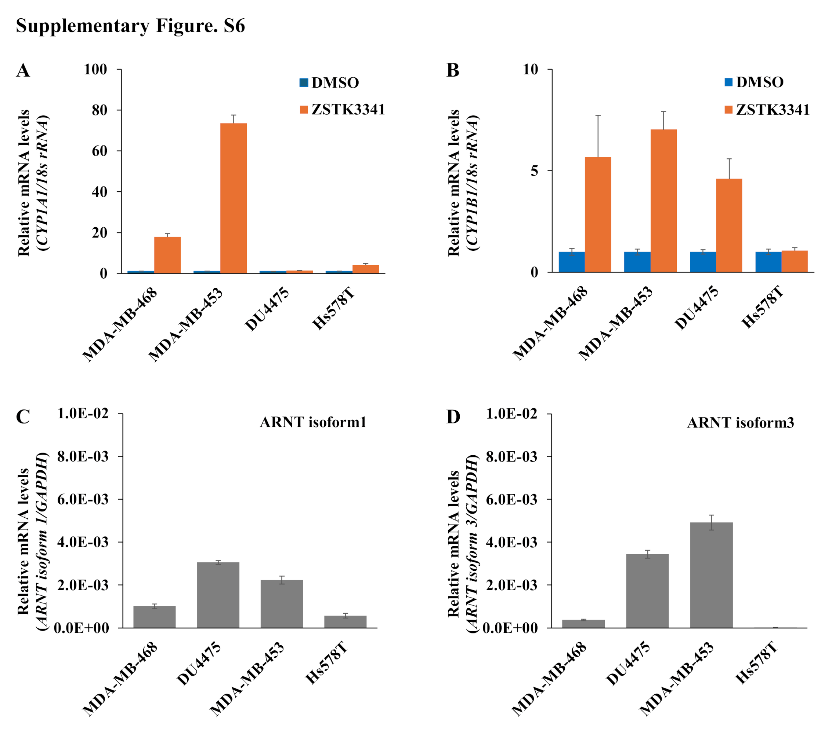


**Supplementary Fig. S6. Evaluation of biomarkers for ZSTK3744**

(A and B) *CYP1A1* (A) and *CYP1B1* (B) mRNA levels were assessed by real-time PCR after treatment with ZSTK3341 (1 μM) for 6 h in MM468, MM453, DU4475, and Hs578T cells. (C and D) *ARNT* isoform 1 (C) and 3 (D) mRNA levels were analyzed in the same cell lines. Sequences of PCR primers are as follows:

ARNT isoform 1/3 forward primer: ACTGCCAACCCCGAAATGAC

ARNT isoform 1 reverse primer: GAGCTCTGCTCATCATCCGA

ARNT isoform 3 reverse primer: TGTGATTTTCCCTGGCAAAC

GAPDH forward primer: GGAGCGAGATCCCTCCAAAAT

GAPDH reverse primer: GGCTGTTGTCATACTTCTCATGG
