# Supplementary material for: ZSTK3744, a Novel Aryl Hydrocarbon Receptor Agonist, Exhibits Efficacy against Chemotherapy-Resistant Triple-Negative Breast Cancer
Source: Cancer Res Commun. 2026 Feb 27;6(2):421–36. doi: 10.1158/2767-9764.CRC-25-0119 (PMC13148475; doi:10.1158/2767-9764.CRC-25-0119)
Supplement: Supplementary Figure S7 — Evaluating the toxicity of AhR agonists in vivo [file crc-25-0119_supplementary_figure_s7_suppsf7.docx]

**
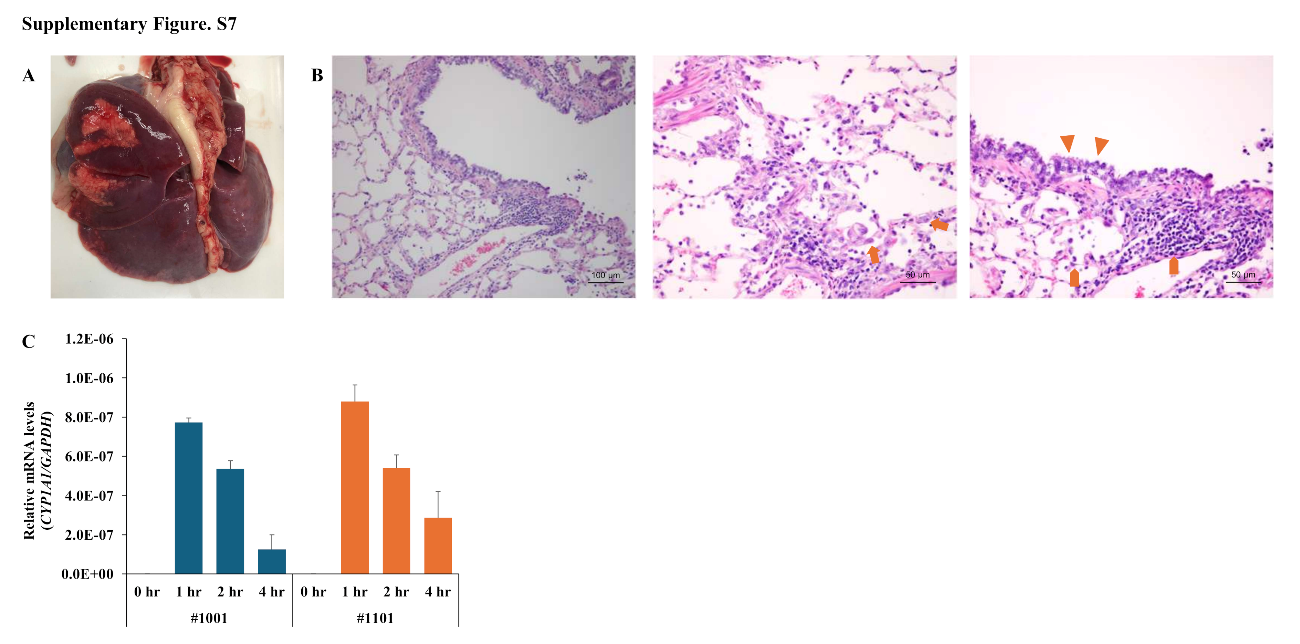
**

**Supplementary Fig. S7. Evaluating the toxicity of AhR agonists *in vivo***

(A) Gross image of the lung immediately after necropsy. A dose of 11.4 mg/kg (228 mg/m²) of AFP464 was administered intravenously once a week to a dog. Respiratory abnormalities were observed the day after the second dose, prompting necropsy. (B) Deparaffinized sections, cut to a thickness of 3 µm, were stained with hematoxylin and eosin for histopathologic examination. In animals administered AFP464, alveolar epithelial regeneration (arrows) with hypertrophy of type II alveolar epithelial cells, bronchial inflammation (five-way arrows), and reactive bronchial epithelial hyperplasia (arrowheads) were observed in the lungs. (C) CYP1A1 mRNA expression levels in the leukocyte-enriched fraction isolated by hypotonic red blood cell lysis after administration of ZSTK3744 (2.4 mg/kg) at the indicated time points, as determined by real-time PCR. #1001 and #1101 represent a male and a female dog, respectively. The TaqMan probe and primer sets for CYP1A1 (Cf03986291_g1) and GAPDH (Cf04419463_gH) were obtained from Thermo Fisher Scientific (Waltham, MA, USA).
