# Supplementary material for: ZSTK3744, a Novel Aryl Hydrocarbon Receptor Agonist, Exhibits Efficacy against Chemotherapy-Resistant Triple-Negative Breast Cancer
Source: Cancer Res Commun. 2026 Feb 27;6(2):421–36. doi: 10.1158/2767-9764.CRC-25-0119 (PMC13148475; doi:10.1158/2767-9764.CRC-25-0119)
Supplement: Supplementary Table S1 — Top 30 significantly upregulated and downregulated differentially expressed genes in MM468/AR cells compared to parental cells [file crc-25-0119_supplementary_table_s1_suppst1.docx]

**Supplementary Table S1. Top 30 significantly upregulated and downregulated differentially expressed genes in MM468/AR cells compared to parental cells**

| Upregulation | | | Downregulation | | |
| --- | --- | --- | --- | --- | --- |
| Gene symbol | Fold changes | p-value | Gene symbol | Fold changes | p-value |
| *ABCB1* | 572.5 | 6.E-10 | *SGCD* | -110.4 | 3.E-05 |
| *CSF3R* | 137.9 | 5.E-06 | *STRA6* | -90.0 | 6.E-06 |
| *GPR87* | 127.2 | 3.E-05 | *GABBR2* | -75.2 | 3.E-05 |
| *CPS1* | 113.6 | 5.E-06 | *SLC28A3* | -59.8 | 3.E-05 |
| *NNMT* | 91.0 | 5.E-06 | *SHISA2* | -49.3 | 8.E-04 |
| *COL12A1* | 81.4 | 5.E-06 | *PLEKHS1* | -45.0 | 1.E-04 |
| *PDZD4* | 73.6 | 7.E-05 | *C2orf72* | -43.5 | 9.E-05 |
| *SERPINB2* | 66.9 | 7.E-05 | *PTPRM* | -42.4 | 1.E-03 |
| *ZDHHC2* | 63.6 | 5.E-05 | *CP* | -40.5 | 4.E-02 |
| *CPQ* | 62.6 | 8.E-05 | *MUC16* | -35.2 | 3.E-04 |
| *PCDHB16* | 59.0 | 6.E-03 | *HSD17B2* | -33.5 | 4.E-04 |
| *HTR7* | 55.4 | 2.E-04 | *ZBED2* | -32.7 | 4.E-03 |
| *COL26A1* | 54.1 | 5.E-04 | *DDC* | -32.5 | 2.E-03 |
| *ZNF765-ZNF761* | 50.5 | 3.E-04 | *ANO1* | -31.8 | 3.E-04 |
| *UGT1A6* | 49.7 | 5.E-05 | *NCAM1* | -30.8 | 5.E-04 |
| *CLMP* | 49.4 | 8.E-05 | *PDZK1* | -30.3 | 6.E-04 |
| *MAT1A* | 47.4 | 3.E-04 | *CAMK4* | -29.3 | 9.E-04 |
| *GULP1* | 47.2 | 8.E-04 | *GPR12* | -29.3 | 3.E-03 |
| *PCDHA1* | 46.7 | 2.E-03 | *IRS4* | -29.0 | 3.E-03 |
| *AOX1* | 43.5 | 9.E-05 | *NEFH* | -27.9 | 6.E-04 |
| *FABP6* | 43.2 | 4.E-04 | *DIPK2A* | -27.8 | 9.E-04 |
| *MAGED1* | 39.9 | 2.E-04 | *NEURL3* | -26.8 | 6.E-04 |
| *MSX1* | 37.0 | 2.E-03 | *COL8A1* | -26.4 | 4.E-03 |
| *CALB2* | 35.4 | 2.E-04 | *A2M* | -25.7 | 1.E-03 |
| *PCDHB8* | 34.7 | 5.E-04 | *COMMD3-BMI1* | -23.8 | 2.E-03 |
| *TP63* | 31.3 | 4.E-04 | *EDN2* | -22.9 | 2.E-03 |
| *PLA2G4F* | 30.5 | 1.E-03 | *CHST9* | -22.0 | 4.E-03 |
| *GPM6B* | 29.6 | 1.E-02 | *SUN3* | -21.3 | 2.E-03 |
| *IL1R2* | 28.9 | 9.E-04 | *KLHL30* | -20.8 | 1.E-02 |
| *C4orf19* | 28.1 | 6.E-04 | *GALNT14* | -20.8 | 6.E-03 |

Total RNA was extracted from parental MM468 and MM468/AR to compare the differential gene expression between parental MM468 and MM468/AR. Comprehensive mRNA expression changes were assessed using RNA-seq. Macrogen Japan Corp. conducted the analysis. The results highlight the top 30 genes that were significantly upregulated or downregulated in MM468/AR cells compared to parental MM468 cells.
