# Supplementary material for: ZSTK3744, a Novel Aryl Hydrocarbon Receptor Agonist, Exhibits Efficacy against Chemotherapy-Resistant Triple-Negative Breast Cancer
Source: Cancer Res Commun. 2026 Feb 27;6(2):421–36. doi: 10.1158/2767-9764.CRC-25-0119 (PMC13148475; doi:10.1158/2767-9764.CRC-25-0119)
Supplement: Supplementary Table S2 — Top 30 significantly upregulated and downregulated differentially expressed genes in MM468/PR cells compared to parental cells [file crc-25-0119_supplementary_table_s2_suppst2.docx]

**Supplementary Table S2. Top 30 significantly upregulated and downregulated differentially expressed genes in MM468/PR cells compared to parental cells**

| Upregulation | | | Downregulation | | |
| --- | --- | --- | --- | --- | --- |
| Gene symbol | Fold changes | p-value | Gene symbol | Fold changes | p-value |
| *ABCB1* | 534.5 | 1.E-09 | *MYEOV* | -204.4 | 1.E-07 |
| *TCEAL8* | 199.9 | 2.E-06 | *ELAPOR1* | -182.8 | 1.E-07 |
| *GULP1* | 179.3 | 1.E-06 | *TVP23C-CDRT4* | -181.7 | 4.E-05 |
| *SLC2A3* | 160.0 | 2.E-07 | *SLC34A2* | -178.7 | 1.E-07 |
| *RORA* | 145.8 | 2.E-06 | *SREK1* | -130.5 | 6.E-07 |
| *COL27A1* | 142.7 | 1.E-06 | *CCL22* | -117.4 | 1.E-06 |
| *DIO2* | 137.8 | 2.E-07 | *PLEKHB1* | -109.3 | 4.E-04 |
| *TSPEAR* | 127.1 | 8.E-07 | *ARL11* | -107.8 | 2.E-05 |
| *ZNF765-ZNF761* | 107.5 | 4.E-06 | *PCDH8* | -102.0 | 2.E-06 |
| *ZDHHC2* | 105.8 | 2.E-06 | *STRA6* | -94.0 | 2.E-06 |
| *COL12A1* | 85.2 | 1.E-06 | *NCMAP* | -86.4 | 1.E-05 |
| *FAT4* | 73.0 | 9.E-06 | *ATP13A5* | -76.0 | 4.E-06 |
| *CPQ* | 70.9 | 3.E-05 | *CD14* | -75.4 | 6.E-06 |
| *NRCAM* | 70.6 | 6.E-05 | *ATP6V0D2* | -75.1 | 3.E-06 |
| *PDZD4* | 66.4 | 4.E-05 | *CLDN10* | -74.4 | 3.E-06 |
| *SERPINI1* | 65.6 | 2.E-05 | *ALDH2* | -72.1 | 5.E-06 |
| *GPR63* | 63.1 | 3.E-05 | *SLC6A14* | -70.6 | 4.E-06 |
| *ZNF275* | 62.7 | 3.E-04 | *TMEM92* | -69.0 | 2.E-04 |
| *KIF26A* | 61.0 | 1.E-04 | *ATP6V1B1* | -67.1 | 4.E-06 |
| *IGSF10* | 59.7 | 7.E-05 | *IQGAP2* | -62.7 | 3.E-04 |
| *JHY* | 58.9 | 4.E-04 | *MAP7D2* | -60.3 | 9.E-05 |
| *CALB2* | 58.6 | 8.E-06 | *ARHGEF6* | -59.1 | 7.E-06 |
| *GALNT18* | 58.0 | 1.E-04 | *CYP4X1* | -58.2 | 8.E-06 |
| *KIF1A* | 53.8 | 2.E-05 | *LRRC55* | -56.4 | 4.E-04 |
| *CFI* | 50.8 | 1.E-04 | *MSMB* | -55.0 | 5.E-04 |
| *SLC12A7* | 48.0 | 1.E-04 | *PRODH* | -53.9 | 2.E-05 |
| *MYH3* | 44.6 | 3.E-04 | *CAPN13* | -49.8 | 3.E-05 |
| *PCDH18* | 43.0 | 5.E-05 | *GPR55* | -48.5 | 2.E-04 |
| *H3C4* | 42.2 | 6.E-04 | *PLEKHS1* | -47.0 | 3.E-05 |
| *ADAMTS5* | 38.4 | 6.E-05 | *ATP6V0A4* | -45.7 | 2.E-05 |

Total RNA was extracted from parental MM468 and MM468/PR to compare the differential gene expression between parental MM468 and MM468/PR. Comprehensive mRNA expression changes were assessed using RNA-seq. Macrogen Japan Corp. conducted the analysis. The results highlight the top 30 genes that were significantly upregulated or downregulated in MM468/PR compared to parental MM468.
